# Decellularized extracellular matrix-based composite scaffolds for tissue engineering and regenerative medicine

**DOI:** 10.1093/rb/rbad107

**Published:** 2023-12-01

**Authors:** Peiyao Xu, Ranjith Kumar Kankala, Shibin Wang, Aizheng Chen

**Affiliations:** Institute of Biomaterials and Tissue Engineering, Huaqiao University, Xiamen, Fujian 361021, PR China; Fujian Provincial Key Laboratory of Biochemical Technology (Huaqiao University), Xiamen, Fujian 361021, PR China; Institute of Biomaterials and Tissue Engineering, Huaqiao University, Xiamen, Fujian 361021, PR China; Fujian Provincial Key Laboratory of Biochemical Technology (Huaqiao University), Xiamen, Fujian 361021, PR China; Institute of Biomaterials and Tissue Engineering, Huaqiao University, Xiamen, Fujian 361021, PR China; Fujian Provincial Key Laboratory of Biochemical Technology (Huaqiao University), Xiamen, Fujian 361021, PR China; Institute of Biomaterials and Tissue Engineering, Huaqiao University, Xiamen, Fujian 361021, PR China; Fujian Provincial Key Laboratory of Biochemical Technology (Huaqiao University), Xiamen, Fujian 361021, PR China

**Keywords:** decellularized extracellular matrix, polymer, bioactive factors, composites, tissue engineering

## Abstract

Despite the considerable advancements in fabricating polymeric-based scaffolds for tissue engineering, the clinical transformation of these scaffolds remained a big challenge because of the difficulty of simulating native organs/tissues’ microenvironment. As a kind of natural tissue-derived biomaterials, decellularized extracellular matrix (dECM)-based scaffolds have gained attention due to their unique biomimetic properties, providing a specific microenvironment suitable for promoting cell proliferation, migration, attachment and regulating differentiation. The medical applications of dECM-based scaffolds have addressed critical challenges, including poor mechanical strength and insufficient stability. For promoting the reconstruction of damaged tissues or organs, different types of dECM-based composite platforms have been designed to mimic tissue microenvironment, including by integrating with natural polymer or/and syntenic polymer or adding bioactive factors. In this review, we summarized the research progress of dECM-based composite scaffolds in regenerative medicine, highlighting the critical challenges and future perspectives related to the medical application of these composite materials.

## Graphical Abstract

**Figure:**
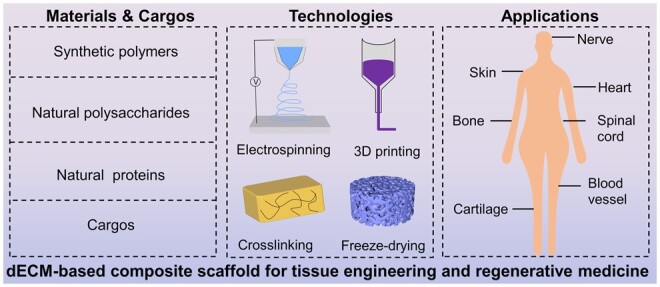

## Introduction

The loss of tissue or organ integrity or function induced by trauma, disease, congenital anomalies and aging are common clinical challenges [1, 2]. Although tissue transplantation is a promising strategy for restoring organ function in patients, severe immune rejection, suitable donor shortages and potential disease transmission remain inevitable challenges, limiting their applicability [3, 4]. To address those challenges, new therapeutical approaches are required to restore damaged tissues or/and organs. In addition, recovering their biofunctions should be addressed to meet the growing demands of patients. Biomaterials have acted as optimal biological substitutes to replace or repair damaged human tissue or organs, providing structural support for cell growth, regulating cellular behavior and mimicking the physical or biochemistry properties of natural tissue [5].

To satisfy clinical requirements, various types of biomaterials, such as organic polymers of synthetic and natural origins, have been widely used as implant scaffolds or wound dressing materials in biomedical fields [6–8]. Using those biomaterials suffers from various limitations, including the potential immunogenic response of natural polymers, the lower biological activity of synthetic polymers and the difficulty in processability of metallic materials [9–11]. With the rapid development in scaffold fabrication processes, composite scaffolds with two or more components have achieved a suitable physical or biological property for tissue engineering based on the synergistic effect [12]. Due to the complex tissue microenvironment, the complex ultrastructure and multiple compositions, and the ongoing reorganization characteristic of natural extracellular matrix (ECM), the design of bioactive scaffold and the fabrication of scaffold with suitable microstructure are still challenging in tissue engineering [13, 14].

Since Poel introduced decellularization in 1948, many studies reported different decellularization technologies to obtain the decellularized ECM (dECM) from cells, tissues or organs [15, 16]. In this context, these decellularization technologies are broadly classified into physical, chemical and enzymatic methods. The physical methods include freeze-thawing, electroporation and mechanical agitation-assisted hydrostatic pressure, among others. To this end, the chemical methods are based on the utilization of detergents, acids/bases, hypotonic/hypertonic solutions, organic solvents and enzymic methods based on DNases and RNases, trypsin to remove cells and maintain the ECM in organs or cells [17, 18]. In addition, different types of dECM have been successfully obtained by supercritical method or the integration of physical, chemical and enzymatic methods [19]. After eliminating the cells and immunogenic factors in decellularization protocols, the native ECM’s original architecture and functional components mainly were preserved [20]. Due to retaining bioactive components and signaling cues, dECM-based biomaterials acted as structures for cell growth and adhesion [21, 22]. They could regulate cell behavior, including cell migration, proliferation and differentiation [23]. More importantly, dECM-based biomaterials have been approved by the Food and Drug Administration (FDA) to fabricate medical products for clinical application, widely investigated as new alternatives for regenerative therapy [24, 25].

Furthermore, the dECM solution could be transformed into hydrogel at an appropriate temperature with three-dimensional (3D) network structures, providing a unique and effective microenvironment for the developing cells and surrounding tissue [26, 27]. However, the unsuitable mechanical rigidity and rapid biodegradability still hinder the application of pure dECM [28, 29]. Bioengineered composite scaffolds composed of dECM with different kinds of biomaterials have provided excellent prospects in regenerative medicine and resulted in hopeful achievements of bioengineered constructions for tissue repair, reconstruction and regeneration. Additionally, considering the requirements of damaged tissue repair, bioactive factors have been utilized effectively as new generations of composite scaffolds to enhance biological activities of dECM.

**Figure 1.**
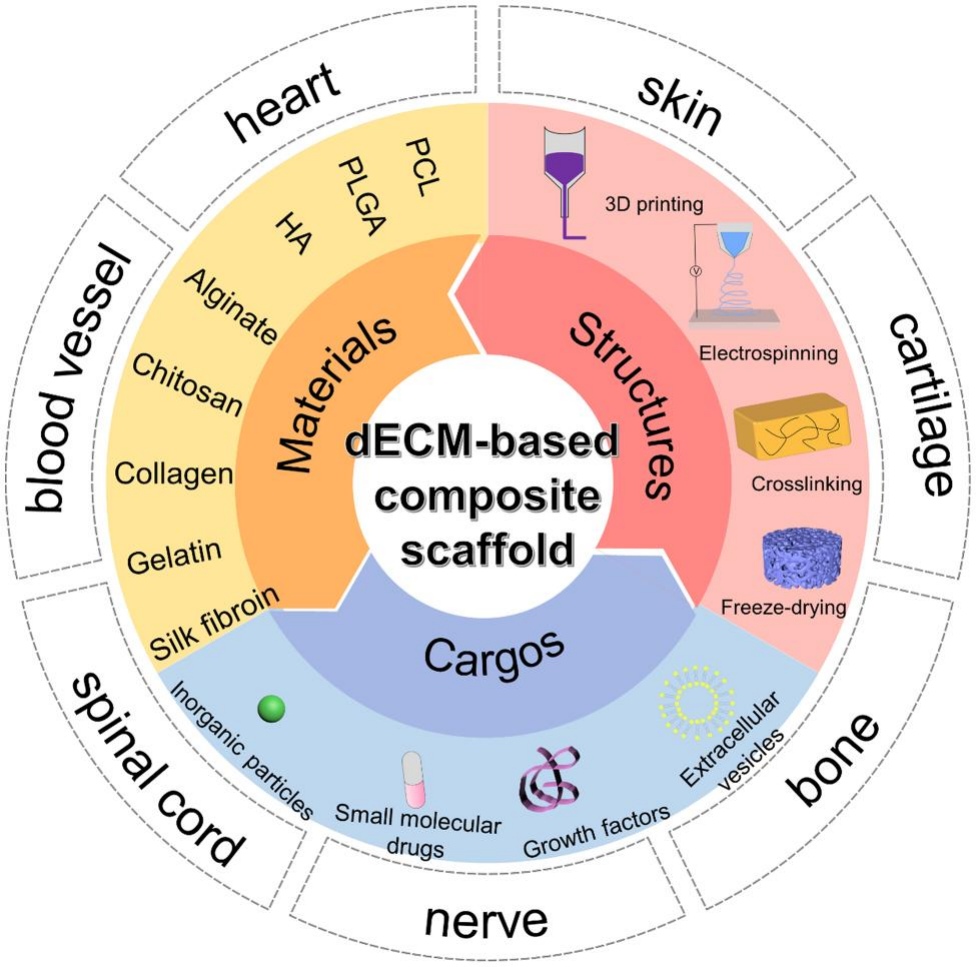
Compositions and applications of dECM-based composite scaffolds for tissue engineering.

In this article, we summarized the recent progress on dECM-based composite scaffolds for tissue regeneration (Figure 1). Firstly, we summed up the contemporary designs of dECM-based composite materials containing nature and synthetic polymers. Considering the synergism of effect in tissue regeneration, the composite scaffolds of dECM combined with bioactive factors are introduced and discussed. Finally, we highlighted the prospects and challenges of dECM-based composite scaffolds and presented insights for developing those composite materials in regenerative medicine.

## dECM–synthetic polymer composite materials

Synthetic polymers with tunable mechanical properties and suitable stability are broadly chosen as fundamental biomaterials for advancing tissue engineering [30]. Moreover, the stable production process, easily reproducibly and ignorable batch difference of those synthetic polymers are sufficient for further clinical and industrial application. Considering the suitable tissue repair requirements, various synthetic polymers have been introduced into prepare dECM-based composite scaffolds with tunable mechanical strength, strong stability *in vivo* and excellent biomimetic properties for application in tissue engineering. In this section, we reviewed the research efforts on some composite scaffolds composed of dECM and synthetic polymers, such as polylactic acid-co-glycolic acid (PLGA), polycaprolactone (PCL), as well as other polymers in tissue regeneration applications. The fabrication method and the application of dECM–synthetic polymer composite materials are summarized in Table 1.

**Table 1.** The fabrication methods and applications of dECM/polymer-based composite scaffolds

Polymer type	Polymer	dECM type	Fabrication method	Main finding	Reference
Synthetic polymer	PCL	Rabbit BMSC	Electrospinning	The composite scaffold could stimulate the chondrogenic	[35]
	PCL	Porcine auricular cartilage	Electrospinning	The composite structure provided a conducive microenvironment for promoting chondrocyte proliferation	[36]
	PCL	Human umbilical cords	Electrospinning	The composite nanofibrous membrane achieved tubular tracheal cartilage reconstruction in a rabbit model	[37]
	PCL	Porcine femoral	Electrospinning	The composite scaffold could promote bone regeneration by increasing osteogenic-related gene expressions	[38]
	PCL	Porcine periosteum	Electrospinning	The core–shell electrospun membrane showed tensile strength and long-term durability with excellent osteogenic properties	[39]
	PCL	Porcine knee menisci	3D printing	The cell-free scaffold displayed favorable biomechanical capacities close to the native meniscus	[43]
	PLGA	Porcine skeletal muscle	Electrospinning and 3D printing	3D-printed scaffold promoted cell orientation and induced myotube formation	[45]
	PLGA	Porcine dermis	3D printing	3D printing bilayer membranous nanofiber inhibited scar hyperplasia by inhibiting collagen fiber deposition and angiogenesis	[46]
	PLGA	Human umbilical cord MSC	3D printing	The 3D-printed scaffold exhibited remarkable bone regeneration capabilities by enhancing the accumulation of M2 macrophages *in vivo*	[47]
	PLGA, PCL	Porcine cartilage	Double emulsion method	The microsphere significantly accelerated chondrogenesis and maintained osteochondral regeneration activity after being incorporated into the filament	[49]
	PLGA	Rat thoracic spinal cord	Electrospinning	The implanted scaffold increased the deposition of collagen and promoted neuronal differentiation	[52]
	PLLA	Porcine sciatic nerves	Electrospinning	The coating of dECM hydrogel accelerated the nerve tissue recovery	[59]
	PLCL	Human adipose tissue	3D printing	This 3D scaffold provided proper mechanical properties and a suitable microenvironment for adipose tissue formation	[61]
	Thiol-Ene PEG	Human placenta	Crosslinking	The composite hydrogel showed tunable storage moduli from 1080 ± 290 to 51 400 ± 200 Pa	[62]
	4-arm-PEG-NHS	Bovine pericardia	Modification	The integrated scaffold showed an anti-adhesion effect after cardiac surgery	[63]
	Genipin-terminated 4-arm-PEG	Porcine urinary bladder, heart, liver, pancreas and small intestine	Crosslinking	The urinary bladder-derived dECM showed the lowest gelation time and the highest shear modulus, exhibiting the most elevated burst pressure	[64]
	Polyurethane	Porcine spinal cord	Freeze-drying	The scaffold showed peripheral nerve regeneration potential in the nerve-transected injury model	[66]
	Polyurethane	Porcine femoral condyles	3D printing	The hierarchical macro–microporous structure almost filled the defect site in *vivo* and exhibited excellent integration to cartilage tissue	[67]
	Polyurethane, PCL	Porcine lateral and medial menisci	3D printing	The controllability bioink with durable architectural integrity provided 3D cell-printed meniscus construction to facilitate tissue regeneration	[68]
	MAP-PNIPAM	Human adipose	Crosslinking	The adipose-derived stem cells-laden hydrogel showed better therapeutic effects in soft tissue regeneration	[70]
Natural polysaccharides	HA-NB	Porcine skin	Crosslinking	The composite hydrogels exhibited strong adhesion to the skin and accelerated irregular wound healing	[75]
	HA functionalized with thiol groups	Porcine joint	3D printing	The layered cartilage scaffold showed bulk compressive and surface mechanics that were similar to the native cartilage	[76]
	HA-MA	Porcine corneal stroma	Crosslinking	The hydrogel showed cornea-matching transparency, slow degradation and good mechanical properties that were suitable for cornea regeneration	[77]
	HA-ADH, HA-CHO	Porcine fat	Crosslinking	The injectable hydrogel provided a tissue-specific microenvironment for resident adipose stromal cells and adipogenic differentiation	[78]
	Sodium alginate, HAT	Bovine aorta	3D printing	The 3D printed construct presented a significant angiogenesis promotion activity and anti-inflammatory responses	[79]
	Alginate	Porcine kidney	Crosslinking	The cell-laden hydrogel showed a potential role in kidney regeneration	[82]
	Alginate	Porcine lung	Crosslinking	3D hybrid scaffold provided a stable environment for achieving the synergistic effect to support neovascularization in airway tissue engineering	[87]
	Alginate	Porcine bone	3D printing	The cell-laden composite microstructure exhibited outstanding osteogenic differentiation activity	[88]
	Alginate	Porcine sciatic nerves	Microfluidics	The gradient microtube based on dECM and alginate guided cell migration and axonal outgrowth for sciatic nerve regeneration	[89]
	Sodium alginate, gelatin	Porcine coronary artery	Crosslinking	The bilayer hybrid scaffold exhibited pore structure and mechanical properties similar to porcine coronary arteries	[90]
	Chitosan	Porcine decellularized nerve tissue	Freeze-drying	The antibacterial scaffold simulated the neural microenvironment for Schwann cells	[95]
	Chitosan	Porcine cardiac extracellular	Crosslinking	The injectable scaffold improved the cardiac function of acute and long-term chronic myocardial infarction *in vivo*	[97]
	Chitosan, PCL	Bovine pericardium	Electrospinning	The composite material showed strong mechanical strength and created a suitable microenvironment for cardiovascular tissue regeneration	[99]
	Oleoyl chitosan	Porcine cancellous bone segments	Crosslinking	The hybrid hydrogel exhibited excellent antimicrobial potential, stronger mechanical strength and showed superior proliferation/differentiation ability	[100]
Natural proteins	Collagen	Bovine eyeballs	Crosslinking	The composite scaffold exhibited a uniform structure with excellent elastic modulus and tensile strength	[104]
	Collagen-MA	Porcine ear, joint, meniscus cartilage and bone	Crosslinking	The composite scaffold reconstructed biphasic cartilage–bone biomimetic microenvironment for osteochondral regeneration	[105]
	Collagen	Porcine brain	Crosslinking	The hydrogel promoted functional recovery in the spinal cord injury model	[106]
	Collagen	Porcine cadaveric join	Crosslinking	dECM solution functionalized scaffold achieved better subchondral bone repair than dECM powder functionalized scaffold	[107]
	Collagen	Rabbit’s joint cartilage	Temperature-induced phase separation	The addition of cartilage dECM with different developmental stages in scaffolds resulted in the different chondrogenic inducibilities with BMSC	[108]
	Gelatin	Rabbit’s femur	3D printing	The 3D scaffold enhanced the mechanical strength, degradation rate and was suitable for bone tissue engineering	[117]
	Gelatin	Human amniotic membrane	Electrospinning	The nanofiber membrane enhanced mechanical properties, and reduced degradation rate, and showed better functional recovery in peripheral nerve injury repair	[118]
	Gelatin, PCL	Porcine knee joint	Electrospinning and freeze-drying	The composite 3D cartilage scaffold with biomimetic microstructure promoted chondrocyte proliferation	[119]
	Gelatin, PLGA	Cow scapular cartilage	Electrospinning and 3D printing	3D-printed fiber-reinforced scaffold enhanced repair articular cartilage effect in rabbits defect model	[120]
	Gelatin, HA-NB	Rabbit auricular cartilage	Crosslinking	The photo-crosslinked hydrogel provided a tissue-specific microenvironment for promoting cartilage repair	[121]
	Gelatin, chitosan	Porcine skin	Freeze-drying method	The biological functional hybrid scaffold showed excellent antibacterial activity for wound healing	[122]
	GelMA	Porcine trabecular	Crosslinking	The scaffold induced human stem cells’ osteogenic differentiation efficiently	[129]
	GelMA	Dental pulp	Crosslinking	Human dental pulp stem cells loaded microcarrier exhibited favorable plasticity and biological performances for endodontic regeneration	[131]
	GelMA	Human placenta	Crosslinking	The photo-crosslinked hydrogel with outstanding mechanical properties could act as a promising material for skin defect healing	[132]
	GelMA	Porcine dental follicles	3D printing	The fabricated scaffold could carry DFUs to produce periodontal modules for subsequent orthotopic transplantation	[134]
	TGUPy	Porcine urinary bladder	Crosslinking	The scaffold showed excellent tissue-adhesive properties, which could be used for abdominal wall defect repair	[135]
	o-Nitrobenzene-modified gelatin	Small intestine submucosa	Crosslinking	The composite hydrogel effectively promoted angiogenesis and collagen deposition for wound healing	[136]
	Silk fibroin	Rat adipose	Crosslinking	The composite hydrogel showed a similar mechanical property to natural adipose tissue	[146]
	Silk fibroin	Porcine knee joint	Customized mold and freeze-drying	The ring-shaped porous scaffold displayed suitable pore size and porosity for cell colonization	[147]
	Silk fibroin, PCL	Human umbilical cord MSC	Electrospinning	The extracellular matrix-modified electrospun fibers could promote axon regeneration of Schwann cells	[148]
	Silk fibroin, PCL	Human amniotic membrane	Electrospinning	The graft provided a native-like mechanical structure and biocompatible microenvironment for cellular infiltration	[149]

BMSC, bone mesenchymal stem cells; Collagen-MA, collagen methacryloyl; DFUs, human dental follicle cells; GelMA, gelatin methacryloyl; HA, hyaluronic acid; HA-ADH, hyaluronic acid grafted adipic acid dihydrazide; HA-CHO, hyaluronic acid grafted aldehyde; HA-MA, hyaluronic acid methacryloyl; HA-NB, nitrobenzene-modified hyaluronic acid; HAT, tyramine-modified hyaluronic acid; MAP-PNIPAM, poly(N-isopropyl acrylamide) grafted mussel adhesive protein; MSC, mesenchymal stem cells; PCL, polycaprolactone; PEG, polyethylene glycol; PLCL, poly(l-lactide-co-caprolactone); PLGA, polylactic acid-co-glycolic acid; PLLA, poly-l-lactic acid; TGUPy, 2-ureido-4[1H]-pyrimidinone unit modified with gelatin; Thiol-Ene PEG, thiol terminated 3-mercaptopropionic acid functioned poly(ethylene glycol); 4-arm-PEG-NHS, 4-arm poly(ethylene glycol) succinimidyl glutarate.

### Polycaprolactone

PCL is a hydrophobic FDA-approved synthetic polymer widely used in biomedical applications [31]. Considering dECM-based scaffolds possess unsatisfactory mechanical properties and rapid degradation, PCL can overcome those problems and combine fascinating characteristics, including prolonged degradation kinetic, and excellent rheological, and viscoelastic properties [32].

A wide range of PCL and dECM-based scaffolds in different forms have been developed using electrospinning techniques, possessing great potential in tissue engineering [33, 34]. For instance, Xu *et al*. [35] cultured chondrocytes on the PCL electrospinning fibrous scaffold, and then obtained the dECM–PCL composite scaffold by decellularization treatment. This composite scaffold was a promising candidate for cartilage regeneration by stimulating the chondrogenic. Feng *et al*. [36] constructed a nanofibrous electrospinning membrane composed of cartilage dECM particles and PCL. Compared with PCL membranes, these hybrid nanofibers with uniform and smooth structures showed excellent mechanical properties and better wettability. The results indicated that the composite structures provided a conducive microenvironment for promoting chondrocyte proliferation. By electrospinning, Xu *et al*. [37] fabricated three membranes with different dECM and PCL ratios and investigated the physicochemical properties. After optimizing the composition *in vivo*, the author constructed a 3D trachea-shaped scaffold based on the composite nanofibrous membrane, which achieved tubular tracheal cartilage reconstruction *in vitro*.

In addition, Dong *et al*. [38] mixed bone dECM and PCL in hexafluoroisopropanol to get a homogenous solution and then obtained nanofibrous scaffolds by electrospinning. The bone dECM and PCL scaffolds showed the enhancement in regulation the osteogenic differentiation of mesenchymal stem cells (MSCs) over the PCL scaffold alone. Overall, the composite scaffold showed the smallest existing defect area in the rat defect model by increasing osteogenic-related gene expressions. Li *et al*. [39] synthesized core–shell composites with periosteal dECM–PCL using co-axial electrospinning. Similarly, Deng *et al*. [40] also designed core–shell structured nanofibers using peripheral nerve dECM modified on the surface of PCL, which could be rolled up into a cylinder-like tube with highly elevated toughness. Compared with PCL-based nanofibers, dECM–PCL-based nanofibers promoted neurite outgrowth, and regulated Schwann cell behaviors. To improve the structural flexibility of PCL scaffolds, Liu *et al*. [41] added silk fibroin directly with PCL and human amniotic dECM to produce bioactive scaffolds. The composite mesh fabricated by electrospinning showed similar mechanical elongation and physiological stiffness compared with the native abdominal wall. Moreover, the hybrid platform inhibited transforming growth factor-β1 (TGF-β1) and collagen expressions in the abdominal wall defects model.

**Figure 2.**
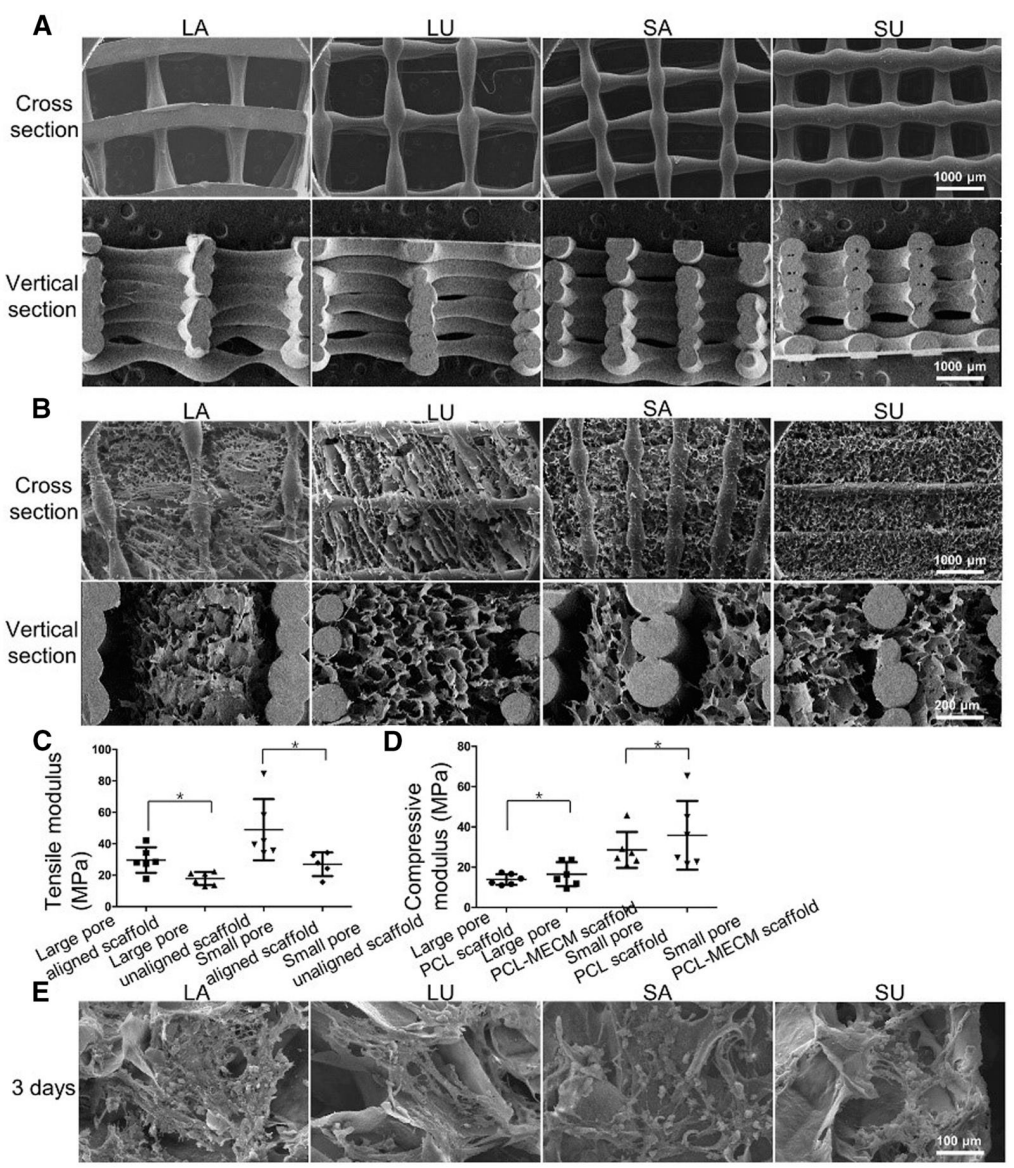
(**A**, **B**) SEM images of PCL and PCL-MECM scaffolds. (**C**, **D**) Biomechanical assessment. (**E**) SEM images of meniscal fibrochondrocytes after 3 days on various scaffolds. Adapted with permission from Ref. [43].

3D printing produced lots of composite scaffolds with customized shapes and structures based on dECM and PCL. Wiggenhauser *et al*. [42] generated a 3D printable scaffold containing dECM and PCL to enhance the cartilage regenerative capacity. Guo *et al*. [43] combined PCL scaffolds with meniscal dECM (PCL-MECM) to produce a composite 3D printing scaffold for simulating the tissue microstructure and environment. Moreover, scaffolds with different pores and structures were constructed to assess the chondrogenic differentiation (Figure 2). Compared to small-pore scaffolds, the collagen type II (COL-II) and SOX 9 expression in large-pore scaffolds were higher after cultured with meniscus fibrochondrocytes. In the rabbit and sheep models, PCL-MECM composite scaffolds protected the tibial plateau from osteoarthritis development and promoted functional neo-meniscus regeneration.

### Polylactic acid-co-glycolic acid

PLGA is a biodegradable polymer approved by the FDA, which was widely applied to regenerate damaged tissues [44]. Lee *et al*. [45] utilized an electrospinning technique to obtain dECM nanofibrous and combined it with a microscale 3D-printed PLGA construct to promote cell orientation and induce myotube formation. Fang *et al*. [46] built a bilayer membranous nanofiber scaffold containing PLGA electrospinning nano nanofiber as the bottom layer and dECM hydrogel as the top layer. This dual-layered scaffold showed a high tensile strength and degradation rate, significantly enhancing the matrix metalloproteinases 1 (MMP-1) and collagen type I (COL-I) expression to inhibit hypertrophic scars formation. Deng *et al*. [47] fabricated PLGA scaffolds and cultured them with human umbilical cord MSC by 3D printing, and then decellularized by physical methods to achieve the sustainable release of proteins in dECM. The 3D-printed scaffolds exhibited remarkable bone regeneration capabilities *in vivo* by enhancing the accumulation of M2 macrophages.

Due to the lower degradation rate, dECM-incorporated PLGA-based scaffolds were used to maintain the stability of dECM and achieve sustained release of bioactive compounds [48]. Ghosh *et al*. [49] loaded cartilage dECM microspheres via double emulsion and then incorporated them in a PCL filament to form 3D scaffolds. The microspheres significantly accelerated chondrogenesis by increasing the COL-II, aggrecan, and SOX 9 gene expressions and maintained osteochondral regeneration activity after being incorporated into the filament. Similarly, Gupta *et al*. [50] prepared novel microspheres based on PLGA for carrying cartilage dECM and bone dECM, which possessed repair tissue effects in the rabbit osteochondral defects model.

**Figure 3.**
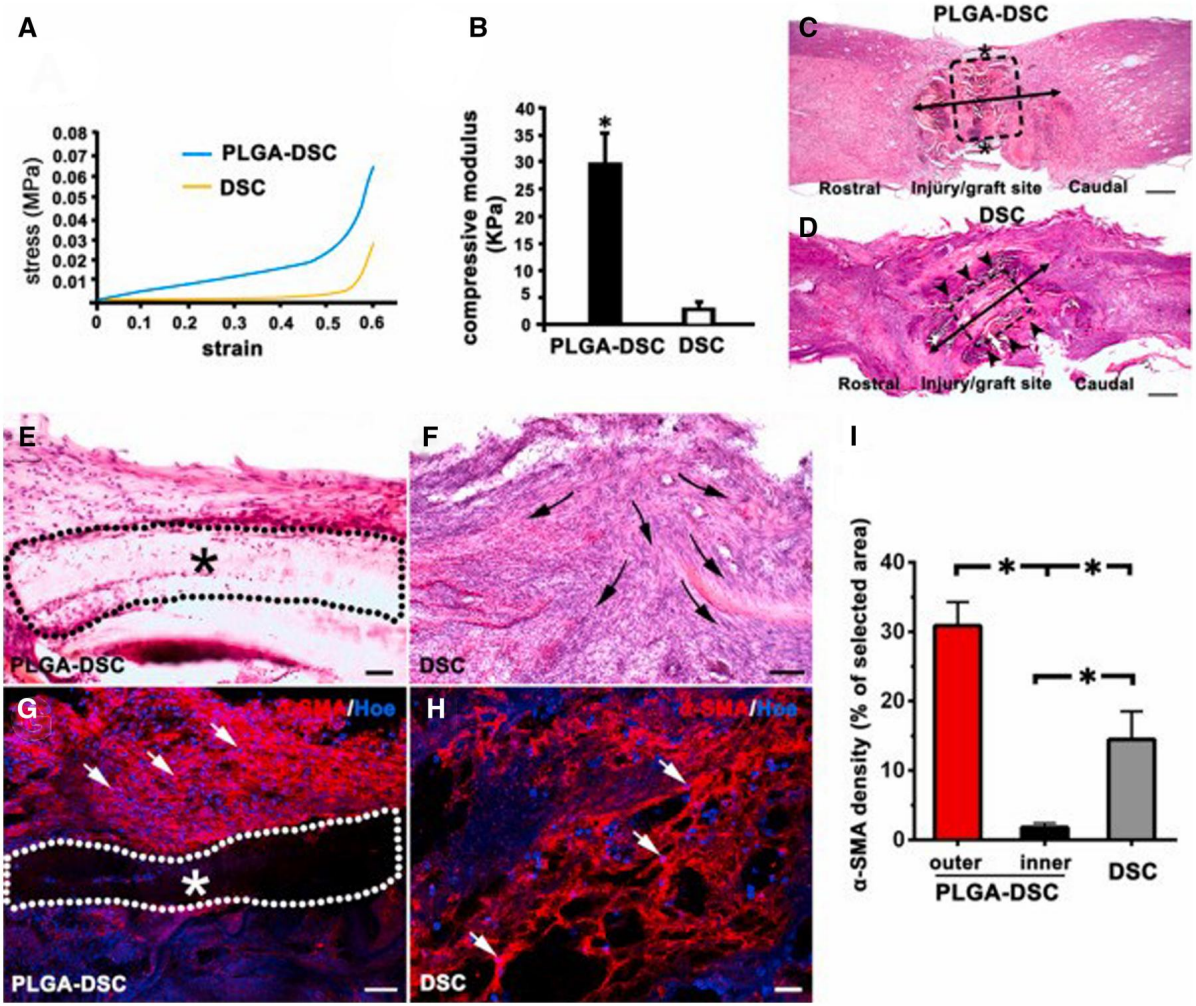
(**A**, **B**) Stress–strain curve and compressive modulus values of PLGA-DSC and DSC scaffolds. (**C**, **D**) H&E staining images of the SCI area at 7 days after implantation. (**E**) High-magnification H&E staining images of PLGA-DSC and (**F**) DSC scaffolds. (**G**) Immunostaining images of the PLGA-DSC and (**H**) DSC scaffolds. (**I**) The quantification of α-SMA staining density. Adapted with permission from Ref. [52].

The combination of PLGA and dECM could also improve the mechanical strength of dECM for cell growth and adhesion support. Lih *et al*. [51] used an ice particle leaching method to prepare a biomimetic scaffold based on kidney dECM incorporated PLGA at different ratios. Enhancing the dECM contents in PLGA scaffolds decreased the compressive moduli and compressive stress, and the composite scaffold with 10% dECM showed a soft-mechanical property for kidney tissue regeneration. In addition, Ma *et al*. [52] obtained spinal cord dECM and coated it by electrospinning to fabricate a mechanically matched scaffold for neural repair. Compared with the compressive modulus of the spinal cord dECM scaffold of 3.2 ± 0.768 kPa, PLGA coated dECM (PLGA-DSC) scaffold was successfully reinforced 10 times with a compressive modulus of 29.8 ± 5.805 kPa. The PLGA-DSC scaffold possessed brilliant mechanical strength and good cytocompatibility, creating a homeostatic microenvironment for neural stem cell migration, residence and differentiation (Figure 3). Moreover, the scaffold showed translational potential to repair function after spinal cord injury with immunoregulation activity.

### Other kinds of polymers

Besides the aforementioned synthetic biomaterials, some synthetic biomaterials with superior biocompatibility and excellent mechanical strength (such as poly-l-lactic acid (PLLA), polyethylene glycol (PEG), poly(l-lactide-co-caprolactone) (PLCL), polyurethane) have also been studied for enhancing the dECM biofunction in tissues engineering [53–58].

For instance, Zheng *et al*. [59] designed PLLA electrospun nanofiber scaffold encapsulated with dECM hydrogel, they analyzed the tissue regeneration effect in rat sciatic nerve defect model, and the post-transplantation results showed that the coating of dECM hydrogel could accelerate nerve tissue recovery than the raw PLLA electrospun scaffold, showing more neurites, and thicker axons thicker myelin. Kim *et al*. [60] reported PLCL and heart dECM-based electrospun nanofibrous for providing a favorable microenvironment conducive to angiogenesis for wound healing. In addition, Lee *et al*. [61] designed a 3D scaffold based on PLCL and human adipose dECM for large-volume fat tissue regeneration application via dual-nozzle 3D printing technique. This 3D scaffold provided proper mechanical properties and a suitable microenvironment for enhancing angiogenesis and adipose tissue formation.

Owing to the facile modifiability and high stability, PEG-based hydrogels for loading dECM or PEG functional dECM hydrogels have shown great promise in medical applications. Fan *et al*. [62] developed a human placenta dECM powder-loaded thiolene PEG-based hydrogel based on UV-induced thiolene click cross-linking reactions. By incorporating dECM at 8 wt%, this composite hydrogel could match the soft tissue moduli, which showed the highest storage moduli at 51 411 ± 199 Pa and exhibited great potential in tissue regeneration. Hashimoto *et al*. [63] functionalized bovine pericardia dECM with four-arm PEG-NHS due to the high excluded interactions with protein. This integrated dECM had flexibility properties and sufficient suturable strength and showed an anti-adhesion effect after cardiac surgery. In another work, Nishiguchi *et al*. [64] synthesized genipin modified with four-arm PEG as a crosslinker to design a dECM-based tissue adhesive. This novel crosslinker was used to combinate with different kinds of dECM hydrogel. The urinary bladder-derived dECM showed the lowest gelation time and the highest shear modulus, exhibiting the most elevated burst pressure.

Polyurethanes is a promising biomaterial for medical applications such as medical device and implantation because of their high mechanical strength, elasticity, fatigue resistance and durability [65]. Wang *et al*. [66] employed polyurethane and spinal cord dECM to build 3D scaffolds, which showed peripheral nerve regeneration potential in the nerve-transected injury model. Chen *et al*. [67] constructed a waterborne polyurethane and cartilage dECM 3D hybrid scaffold with a hierarchical macro–microporous structure. The addition of dECM not only significantly enhanced the porosity of this scaffold but also improved the expression of COL 2A1, SOX 9 and ACAN was significantly increased after being cultured with adipose-derived stem cells at 14 days than polyurethane scaffold. After subcutaneous implantation with cartilage defect in a rabbit model for 3 months, the polyurethane–dECM scaffold almost filled the defect site *in vivo*, it exhibited excellent integration with surrounding cartilage tissue. In addition, Chae *et al*. obtained different kinds of dECM bioink for cell-loading and printed on the polyurethane and PCL frame. This composite scaffold with excellent mechanical properties provided an appropriate biochemical microenvironment for regulating cell behavior [68, 69]. Jeon *et al*. [70] designed poly(N-isopropyl acrylamide) grafted mussel adhesive protein (MAP-PNIPAM) and incorporated it with adipose dECM powder to form hydrogel network. Moreover, the hydrogel with injectable and tissue adhesive activity could promote the integration of adipose-derived stem cells in soft tissue defect sites for long term.

## dECM–natural polymer composite materials

In contrast to synthetic polymers, natural polymers are regarded as effective biomaterials to promote tissue repair with higher biocompatibility, sufficient biodegradability and lower toxicity. Considering the exciting results from dECM-based scaffolds, natural polymers were used to obtain new-generation functional composite scaffolds by combining with dECM for tissue regeneration. Therefore, the recent developments of composite scaffolds formed by the combination of natural polysaccharides (alginate, hyaluronic acid (HA) and chitosan) and natural proteins (collagen, gelatin and silk fibroin) with dECM will be discussed. The applications of dECM–natural polymer composite materials are also summarized in Table 1.

### Natural polysaccharides

#### HA derivatives

HA is a natural hydrophilic polysaccharide, an important ECM component around cells and tissue [71]. Due to the essential biofunction in the tissue regeneration process, HA has been widely studied as a component of scaffold, such as hydrogels, sponges and films. Although HA could not form hydrogels alone, HA derivatives based on chemical reactions have been introduced in HA-based hydrogel to achieve a synergistic effect with dECM [72–74].

**Figure 4.**
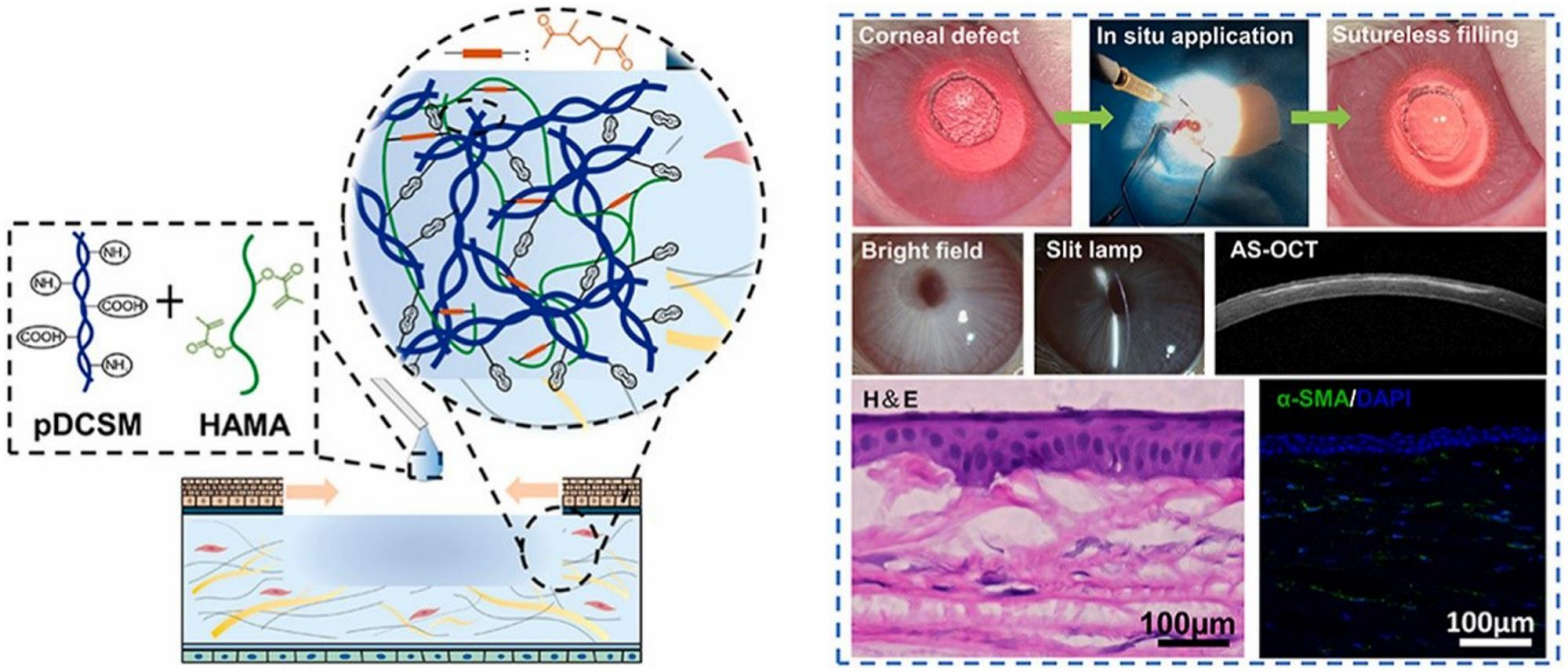
Schematic illustrations and results of the hybrid hydrogel applied onto corneal defect *in situ* for long-term regenerative repair. Adapted with permission from Ref. [77].

Among the crosslinking methods, photo-crosslinking is a highly versatile method that could produce hydrogel by controlling crosslinking time and strength using light. Bo *et al*. [75] reported a photo-crosslinking hydrogel composed of nitrobenzene-modified HA (HA-NB) and dECM. Human adipose-derived MSCs were loaded into the composite hydrogel system, exhibiting more satisfactory therapeutic effects than other groups in irregular wounds. Barthold *et al*. [76] functionalized HA with thiol groups to form a disulfide bond-related hydrogel network with dECM particles and used it as a bioink to construct a 3D hydrogel with high biocompatibility. In another study, Shen *et al*. [77] reported a dual-crosslinked hybrid hydrogel by using methacrylated HA (HA-MA) and corneal stroma dECM (Figure 4). The dual-crosslinked composite hydrogel exhibited not only high transparency with a stable structure but also showed excellent force-resistance and slow degradation under enzyme environment. *In vitro* results showed that dual-crosslinked hydrogel significantly enhanced keratocan and lumican expressions and decreased the expression of myofibroblast-related genes compared to tissue culture plates. Moreover, the implantation of HA-MA/dECM scaffolds in corneal defect model greatly reduced corneal scarring and promoted the stromal wound healing process, providing great potential for long-term corneal tissue regeneration.

Lin *et al*. [78] used HA-aldehyde (HA-CHO) and adipic acid dihydrazide grafted HA (HA-ADH) as raw materials to incorporate fat dECM. This injectable composite hydrogel provided a better microenvironment for loading adipose stromal cells and including adipogenic differentiation. Isik *et al*. [79] constructed perfusable dECM-loaded hydrogel with high adhesion, self-healing properties and enhanced bioactivity, which were formed by the fast gelation of sodium alginate with tyramine-modified HA. By controlling the composition ratios of hydrogel and gelation induce factors, a 3D scaffold with impressive mechanical robustness and elasticity for cell growth. Furthermore, the printed construct presented a significant angiogenesis promotion activity and anti-inflammatory responses, evidencing the potential in cardiovascular surgery application.

#### Alginate

Alginate, a naturally available polysaccharide obtained from seaweeds, provides remarkable biocompatible and biodegradable characteristics, particularly interesting for biomedical applications [80, 81]. Alginate-based materials with different forms have been utilized with dECM to mimic the tissue microenvironment [82–84]. Curley *et al*. [85] improved the mechanical properties of dECM hydrogel by mixing alginate and heart dECM, which showed storage modulus of 1.6 kPa and compressive modulus of 29 kPa. Park *et al*. [86] optimized the bioink composition based on alginate and heart dECM for cell bioprinting, which provided a cell-friendly microenvironment for cell growth and maturation promotion.

Due to alginate properties, De Santis *et al*. [87] designed alginate and dECM-based bioink to build hybrid human airways tissue engineering scaffolds by 3D printing. *In vivo* results demonstrated that this hybrid scaffold provided a stable environment to achieve the synergistic effect for supporting neovascularization with a lower inflammatory response. In another work, Lee *et al*. [88] used methacrylate-modified dECM (dECM-MA) and mixed it with sodium alginate as bioink to 3D scaffold fabrication, which induced excellent osteogenic activities *in vivo*. Additionally, Jin *et al*. [89] generated a microtube containing sciatic nerve dECM and alginate using microfluidic technology via polyvinyl alcohol solution as sacrificing core. The microtube with a homogeneous core–shell structure and controllable gradient encapsulation was successfully obtained by controlling the component and flow ratio of the fluid. Moreover, this composite microtube enhanced the Schwann cell proliferation and migration for nerve repair and also inhibited muscular atrophy *in vitro*. Similarly, Du *et al*. [90] reported a bilayer hybrid scaffold that made of alginate and gelatin mixed hydrogel coated on the surface of coronary artery dECM. The core–shell scaffold possessed a pore structure and showed similar mechanical properties to native porcine coronary arteries, which provided a suitable environment for cell proliferation.

#### Chitosan

Chitosan is an abundant natural polysaccharide obtained from chitin, widely exists in crustacean shells [91]. Owing to the advantages, such as antibacterial, biodegradable, anti-inflammatory and biocompatible, the chitosan-based scaffolds have broad medical applications in tissue regeneration and repair [92–94]. Kong *et al*. [95] constructed porous nerve dECM–chitosan scaffolds by freeze-drying technique, exhibiting excellent antibacterial activity, and simulated the neural microenvironment for Schwann cells. Similarly, Ye *et al*. [96] designed a dECM-covered chitosan globule for bone regeneration. In another study, Efraim *et al*. [97] prepared a dECM injectable scaffold with different amounts of chitosan for acute myocardial infarction treatment, which provided mechanical support during cardiac tissue repair. The hemodynamics results showed that this composite scaffold could improve cardiac function and alleviate damage and myocardial infarction after treatment.

Combining chitosan with other polymer or chitosan derivative-based scaffolds, such as hydrogels, membranes and sponges, has attracted significant attention for loading dECM [98, 99]. For instance, Datta *et al*. [100] prepared a novel biohybrid hydrogel using fatty acid-modified chitosan and bone dECM to deliver human amnion-derived MSCs. The composite hydrogel demonstrated high mechanical strength, excellent antimicrobial potential and superior cell proliferation activity. Furthermore, the hydrogels significantly improved the osteogenic-related gene expressions, including COL-I, osteocalcin (OCN) and osteopontin (OPN). The cell-laden hydrogel positively impacted newly formed bone tissues with surrounding tissues at the defect site. Chu *et al*. [101] prepared chitosan and gelatin-based composites spongy scaffold with various compositions and then modified with liver dECM. The optimized spongy scaffold demonstrated a great blood absorption rate and fast blood clotting, demonstrating good application potential as potential hemostatic material.

### Natural proteins

#### Collagen

Collagen is the most abundant protein in vertebrate animals and a prevalent component found in ECM [102]. Moreover, collagen could promote cell adhesion and migration, as well as regulate cellular growth, which plays a dominant role in maintaining ECM homeostasis [103]. Due to its abundance source and facility of collagen-based scaffold, the combination with dECM and collagen composite scaffold also attracted lots of attention from researchers. For instance, Hong *et al*. [104] mixed compressed collagen with cornea dECM to construct a dense collagenous scaffold for mimicking biomimetic corneal stroma analog. Due to the reduction of fibril diameter, the addition of cornea dECM significantly improved the optical transparency of the collagen scaffold and maintained the native keratocyte morphology and functions. Hua *et al*. prepared a photo-cross-linkable cartilage–bone integrated hydrogel using methacryloyl derivative (collagen-MA) and cartilage dECM-MA hydrogel. Then, they incorporated bone dECM particles to effectively promote osteochondral regeneration [105]. The bone MSC (BMSC) laden integrated scaffolds that contain bone and cartilage dECM induced chondrogenesis in the early stage and initiated bone regeneration. *In vivo* studies on rabbits with osteochondral defects demonstrated that this composite scaffold could achieve satisfactory cartilage–bone interface integration.

**Figure 5.**
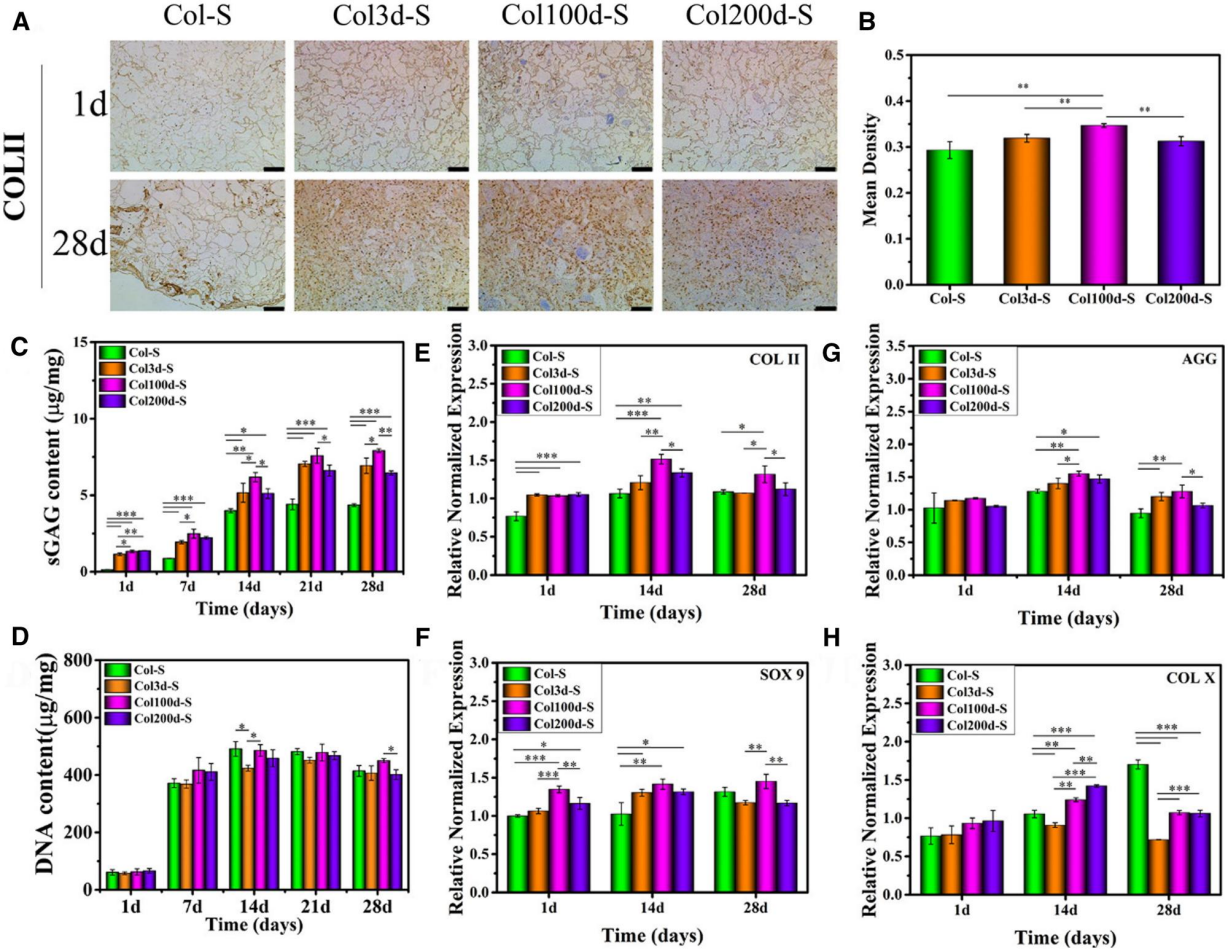
(**A**) The immunohistochemistry staining, (**B**) the semiquantitative analysis of COL-II after culturing BMSC on various scaffolds. Quantitative determination of (**C**) GAG and (**D**) DNA. The chondrogenic genes expression of (**E**) COL-II, (**F**) SOX 9, (**G**) AGG and (**H**) COL X. Adapted with permission from Ref. [108].

Researchers also discussed the tissue regeneration effect of collagen-based scaffold combined with dECM at different ratios, tissue types or forms. Hong *et al*. produced collagen-based hydrogels that contained various concentrations of dECM (1, 5 and 8 mg/ml) [106]. Adding dECM could enhance the cortical neuron's length, whereas the length of hippocampal neurons was improved in dECM concentration at 5 and 8 mg/ml. Lu *et al*. [107] fabricated collagen scaffolds that functionalized cartilage dECM in solution and particle forms. Compared with dECM particle-functionalized scaffolds, the growth factors were more dispersed in dECM-functionalized scaffolds, thus facilitating the BMSC’ proliferation and chondrogenic differentiation. More importantly, the dECM composite scaffold induced the neocartilage tissue formation on the surface of articular, and the dECM solution functionalized scaffolds achieved neocartilage formation with better structure and smoother surface after implantation. Cao *et al*. [108] obtained rabbit articular dECM cartilage at 3, 100 and 200 days (Col 3d-S, Col 100d-S and Col 200d-S) and crosslinked with collagen to investigate the chondrogenic inducibilities. The degradation rate result of collagen–cartilage dECM scaffolds indicated the cartilage dECM with higher matured could prolong the degradation time of collagen–cartilage composite scaffold. Moreover, *In vivo* and *vitro* results demonstrated that Col 3d-S efficiently accelerated subchondral bone regeneration, while Col 100d-S exhibited exceptional chondrogenic performance (Figure 5).

#### Gelatin

Gelatin is an essential protein obtained from native collagen by hydrolysis, which can be obtained by disrupting the intermolecular bonds and breaking the α-helix conformation of collagen [109, 110]. Compared with collagen, gelatin maintains the RGD (Arg–Gly–Asp) sequence to promote cell adhesion and also shows lower immunogenicity [111]. Due to their biocompatibility and acceptable toxicity, gelatin-based medical products (such as Cutanplast, Surgiflo and Floseal) have been used for hemostasis in surgeries [112]. Moreover, gelatin can act as an excellent candidate to produce biomedical materials, such as hydrogels, scaffolds or films for promoting tissue repair [113, 114]. Thus, gelatin is also commonly used to fabricate hybrid scaffolds with dECM for tissue generation [115, 116]. For instance, Kara *et al*. [117] fabricated bone dECM particle-reinforced gelatin as bioink to build scaffold for mimicking the bone tissue. Compared with pure gelatin 3D scaffold, this composite scaffold increased the roughness of the scaffold surface and improved the elastic modulus. Chen *et al*. [118] integrated the gelatin nanofiber membrane into the dECM membrane based on the interfacial covalent bonding to maintain the biological activity of dECM. *In vitro* results showed that this hybrid membrane enhanced the adhesion and proliferation of Schwann cells and macrophage polarization. Moreover, implanting gelatin/dECM membrane greatly promoted axon regeneration and myelination and improved muscle function in a rat model.

Moreover, some researchers have paid attention to the hybridization of gelatin with other biomaterials to achieve improved mechanical properties or other performances. For example, Li *et al*. [119] indicated that the gelatin/PCL nanofiber in the dECM scaffold could increase its mechanical properties and structural stability. The results proved that combining electrospun nanofibers and a dECM-based scaffold could promote chondrocyte proliferation and facilitate early maturation of the cartilage. Similarly, Chen *et al*. [120] prepared electrospinning fiber based on gelatin and PLGA and then mixed it with dECM to form a 3D scaffold. The histological results after implantation for 12 weeks demonstrated that the electrospinning fiber-assisted 3D scaffolds improved the outcomes of cartilage repair *in vivo*. Xu *et al*. [121] designed a photo-crosslinked hydrogel system consisting of HA-NB/gelatin/dECM for loading chondrocytes. The composite system showed satisfactory mechanical properties, and the addition of dECM demonstrated enhanced cartilage regeneration activity. Xu *et al*. [122] added chitosan into dECM/gelatin scaffolds, which exhibited high porosity, appropriate elastic modulus, and degradability for wound healing. Moreover, the chitosan imparted hybrid scaffolds with high antibacterial properties.

**Figure 6.**
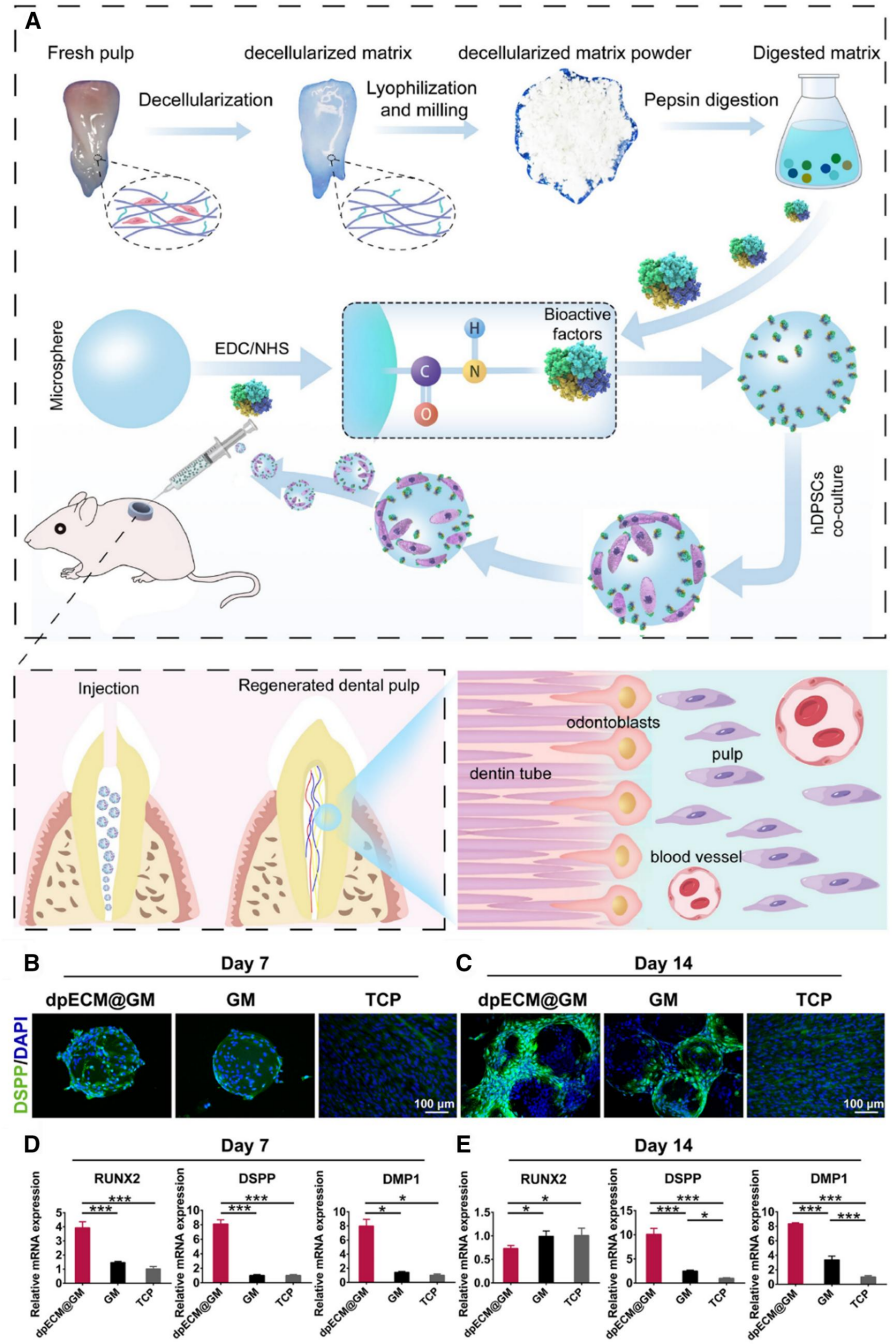
(**A**) Schematic diagram showing the fabrication of hydrogel microspheres functionalized with dental pulp dECM and their application in regenerative endodontic treatment. (**B**, **C**) Dentin phosphophorym immunofluorescence analysis of hDPSCs. (**D**, **E**) Odontogenic-related gene mRNA expression levels. Adapted with permission from Ref. [131].

Due to the poor mechanical strength and mismatched degradation of the gelatin-based scaffold, the chemical modification of gelatin with methacryloyl (GelMA) has been increasingly developed [123, 124]. GelMA maintained functional sequences of gelatin (Arg–Gly–Asp and the matrix metalloproteinase) and could form hydrogel with tunable physicochemical properties by photo-crosslinking [125]. The GelMA-combined dECM scaffold with different forms has shown extraordinary behaviors in regenerating various tissues [126–128]. For instance, Gao *et al*. [129] incorporated bone dECM particles into GelMA hydrogel for bone defect reconstruction. With increasing the dECM content, the compressive strength and stiffness of the hydrogel were also improved. He *et al*. [130] prepared spinal cord dECM and GelMA hydrogel and achieved a synergistic effect with human menstrual blood-derived stem cells for spinal cord injury treatment. Zheng *et al*. [131] developed GelMA microspheres and conjugated dental pulp dECM to mimic pulp-specific microenvironment (Figure 6). The multifunctional hydrogel microspheres could maintain the activity and ability proliferation of human dental pulp stem cells (hDPSCs). Notably, after cultured the cells in scaffold for 14 days, these composite microspheres significantly enhanced the expression of dentin phosphophorym and dentin matrix protein1 and reduced the runt-related transcription factor 2 in hDPSCs compared with other groups. Moreover, the addition of dECM in this multifunctional hydrogel microspheres also resulted in the odontogenic, angiogenic and neurogenic differentiation of cells. In addition, Zhang *et al*. [132] designed a visible light cross-linkable hydrogel based on MA-modified human placenta dECM and GelMA for wound healing. Besides enhancing the tensile strength, the cross-linkable hydrogel also possessed water-absorption capacity. Moreover, the hybrid hydrogel could support the proliferation of fibroblasts by simulating the extracellular microenvironment.

Recently, 3D printing techniques have been introduced to fabricate dECM and GelMA composite hydrogel for mimicking the 3D microstructure in tissue engineering. Chen *et al*. [133] fabricated GelMA and dECM-based scaffolds to understand the influence of tensile stimulation on chondrocytes. Yang *et al*. [134] prepared a 3D biomimetic periodontal module that containing GelMA, dECM and human dental follicle cells (DFUs). *In vivo* results demonstrated that the fabricated 3D biomimetic periodontal module could promote fibrogenesis and osteogenic of DFUs, and could carry DFUs to produce periodontal modules for subsequent orthotopic transplantation. Moreover, the study in periodontal defect of a beagle model confirmed their effectiveness in alveolar bone recovery, which was highly desirable for periodontal regeneration.

To further improve the tissue-adhesive activity, Nishiguchi *et al*. [135] functionalized gelatin with the 2-ureido-4[1H]-pyrimidinone unit (TGUPy) and mixed it with urinary bladder dECM to obtain a composite patch. Compared to the dECM patches, TGUPy–dECM composite patch enhanced the strain (8-fold) and stress (10-fold) that did not influence the porous structures and swelling behavior, showing an outstanding tissue-adhesive property, which could be used for abdominal wall defect repair. In another work, Wang *et al*. [136] designed o-nitrobenzene-modified gelatin coated with dECM hydrogel as a multifunctional wound dressing. *In vivo* experiments of the rat model demonstrated that the composite hydrogel effectively promoted angiogenesis and collagen deposition, indicating its good effect for accelerating epidermal regeneration in the wound healing process.

#### Silk fibroin

Compared to collagen and gelation-derived biomaterials, silk fibroin purified from silkworm cocoons has achieved superior mechanical performance [137]. In recent years, owing to the excellent biocompatibility, tunable degradability and abundant sources of silk fibroin, silk fibroin-based bio-scaffolds have received considerable attention for tissue regeneration [138, 139].

Based on the unique propriety, the silk fibroin–dECM composite scaffold with different shapes was constructed by previous studies, which provided a new approach to tissue regeneration [140, 141]. Liu *et al*. [142] cultured cardiac fibroblasts in silk fibroin scaffold and decellularized to obtain the cell dECM-coated scaffold to mimic the myocardial microenvironment. Gholipourmalekabadi *et al*. [143] designed electrospun nanofibrous silk fibroin that coated with human amniotic dECM membrane to seeded adipose-tissue-derived MSC, which could accelerate wound healing process regeneration and significantly reduces scars formation in burn wound. Dhasmana *et al*. [144] modified silk fibroin on the acellular dermal matrix scaffold to prolong the degradation time and lower immune response in wound healing.

Zhu *et al*. [145] fabricated a hybrid nanofiber scaffold silk fibroin and dECM using the electrospinning method for islet transplantation. Kayabolen *et al*. [146] prepared silk fibroin/dECM matrix hydrogel with different ratios via crosslinking to mimic the native tissue microenvironment. The composite hydrogel with similar mechanical properties to natural adipose tissue promoted the vascularization process for adipose tissue engineering. Gao *et al*. [147] fabricated silk fibroin and dECM scaffold with a ring-shaped porous structure via freeze-drying method. The hybrid scaffold displayed suitable water absorption, degradation rate and proper mechanical strength, which accelerated the chondrogenesis by enhancing the cartilage-related expression of SOX9, ACAN and COL-II. Moreover, the BMSC-embedded silk fibroin–dECM scaffold demonstrated excellent cartilaginous ring regeneration activity after subcutaneous implantation for 4 weeks, along with comparable mechanical strength and components to the normal trachea in a rabbit model.

**Figure 7.**
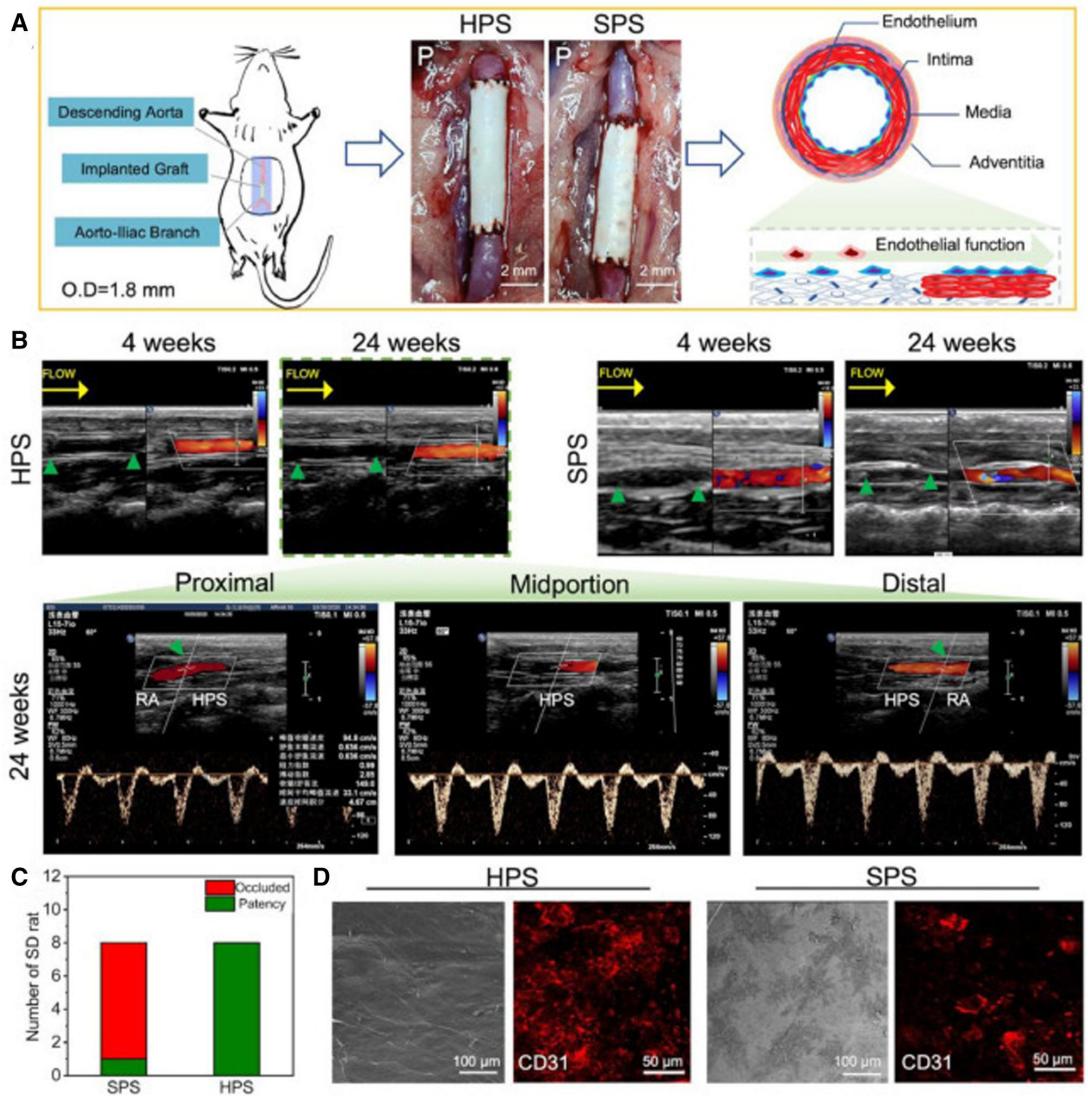
(**A**) Schematic illustration of a vascular graft implanted into the descending abdominal aortas of Sprague–Dawley rats. (**B**) Color doppler ultrasound images after implantation. (**C**) The patency analysis. (**D**) SEM and CD31 immunofluorescent staining images of the luminal surface of the vascular grafts. Adapted with permission from Ref. [149].

In addition, other polymers have been combined with silk fibroin and dECM-based scaffolds for tissue engineering [148]. Typically, Liu *et al*. [149] constructed human amniotic membrane dECM-based grafts with tubular structures and rolled with PCL/silk fibroin nanofiber to form the luminal surface. The core–shell graft integrating dECM with PCL/silk fibroin exhibited better endothelial cell proliferation, inhibition of collagen secretion and suitable mechanical strength, remodeling the similar native structure over 24 weeks of implantation (Figure 7). In another work, Lee *et al*. [150] developed a 3D porous scaffold that consisted of collagen, dECM and silk fibroin using low-temperature printing technology. Moreover, the porous composite scaffold showed highly improved compressive modulus for hard tissue regeneration.

## dECM–bioactive factor composite materials

Combining scaffold fabrication technologies, the bioactive factors are incorporated into biomaterials to create a multifunctional cell-supporting structure to show a better tissue repair effect than a single scaffold due to the synergistic effect. A wide range of bioactive factors, such as growth factors, small molecular drugs, inorganic particles and bioactive factors are obtained for cells, have been combined with the dECM scaffold to promote damaged tissue or organ healing. The application in tissue engineering of bioactive factors loaded dECM-based scaffolds are summarized in Table 2.

**Table 2.** Bioactive factor loaded dECM-based composite scaffolds for tissue application

Scaffold	dECM type	Bioactive factor	Applications	Reference
dECM	Calf nose	IGF-1	Cartilage regeneration	[152]
dECM	Rabbit skeletal muscle	IGF-1	Muscle regeneration	[153]
dECM	Porcine heart	Stromal cell-derived factor 1	Myocardial infarction treatment	[154]
dECM	Porcine spinal cord	bFGF	Spinal cord injury treatment	[155]
dECM	Rat brain	bFGF	Parkinson’s disease treatment	[156]
Chitosan, dECM	Rabbit annulus fibrosus	bFGF	Annulus fibrosus tissue engineering	[157]
PCL	Rabbit adipose	bFGF	Breast tissue engineering	[158]
Aptamer-functionalized GelMA, dECM, dECM	Porcine articular cartilage	TGF-β3	Cartilage regeneration	[160]
dECM	Osteogenic-differentiated human MSC	BMP-2	Bone regeneration	[161]
dECM	Porcine small intestine	BMP-2	Bone defect regeneration	[162]
PEGDA, dECM	Porcine spine	TGF-β1	Promote annulus fibrosus repair	[163]
Silk fibroin, dECM	Goat articular cartilage	TGF-β3	Cartilage tissue engineering	[164]
PCL, GelMA	Sheep knee	TGF-β3	Cartilage regeneration	[165]
Silk fibroin, dECM	Goat cartilage and bone	TGF-β, BMP-2	Osteochondral repair	[166]
PEGDA, dECM	Porcine meniscus	TGF-β1, BMP-7 and IGF-1	Cartilage regeneration	[167]
PCL	Rat abdominal artery	Heparin, VEGF	Vascular grafts fabrication	[168]
dECM	Porcine adipose	VEGF	Enhancing angiogenesis	[169]
Silk fibroin, dECM	Porcine small intestinal submucosa	VEGF and TGF-β1	Vascular tissue engineering	[170]
HA-MA, dECM	Porcine aortas	VEGF and HGF	Cerebral angiogenesis promotion	[171]
dECM	Goat small intestine	Curcumin	Wound healing	[173]
dECM, PCL	Porcine skin	Usnic acid	Wound healing	[174]
dECM, decellularized kelp	Porcine liver	Coumaric acid	Wound healing	[175]
GelMA, dECM	Porcine tendon tissues	Asiaticoside	Wound healing	[177]
dECM	Bovine pericardia	Copper@tea polyphenol nanoparticles	Wound healing	[178]
dECM	Human adipose	Simvastatin, hydroxyapatite	Bone regeneration	[179]
Silk fibroin methacryloyl, dECM	Porcine skeletal muscle	Quercetin	Muscle regeneration	[180]
HA, dECM	Porcine stomach	Omeprazole	Repairing gastric ulcer	[181]
PLGA, dECM	Porcine small intestinal submucosa	Pifithrin-alpha	Bone regeneration	[182]
PGCL	BMSC	Icariin	Bone regeneration	[183]
dECM/oleoyl chitosan	Porcine bone	Alendronate and BMP-2	Bone regeneration	[184]
HA-heparin	Human great saphenous vein	Heparin	Vascular grafts	[185]
Oxidized chondroitin sulfate	Porcine aortas	Adenine	Vascular regeneration	[186]
Alginate, dECM	Porcine aortic tissue	Atorvastatin	Ischemic diseases treatment	[187]
PLCL	Porcine thoracic aorta	Salidroside	Vascular regeneration	[189]
PCL	Rat aorta	Rapamycin	Vascular regeneration	[188]
dECM	Bovine achilles and neck tendons	Hydroxyapatite	Bone regeneration	[193]
PCL, dECM	Bovine small intestinal submucosa	Hydroxyapatite	Bone regeneration	[194]
PLLA, dECM	Porcine thigh bone	Hydroxyapatite	Bone regeneration	[195]
dECM	Porcine small intestine	Sr^2+^/Fe^3+^ co-doped hydroxyapatite	Bone regeneration	[196]
dECM	Porcine femoral condyle	Graphene oxide	Cartilage regeneration	[198]
dECM	Human placental and umbilical cord	Graphene oxide	Vascular graft	[199]
dECM	Rat liver	Nano-graphene oxide	Liver regeneration	[200]
dECM	Porcine adipose	Reduced graphene oxide	Neural tissue engineering	[201]
dECM	Porcine myocardial	Reduced graphene oxide	Heart tissue engineering	[202]
PLGA, dECM	Porcine liver	Magnesium-enriched graphene oxide nanoscrolls	Bone regeneration	[203]
Chitosan, dECM	Rat skin	Carbon nanodot	Wound healing	[204]
dECM	Goat small intestine submucosa	Nanoceria	Skin tissue engineering	[205]
dECM	Goat small intestine submucosa	Nanoceria, curcumin	Wound healing	[206]
GelMA, dECM	Fish scale	Black phosphorus	Bone defect	[209]
PLGA, dECM	Porcine liver	Copper oxide nanozymes	Acute liver failure treatment	[210]
dECM	Fish scale	Osteogenic BMSC exosomes	Bone regeneration	[214]
dECM	Porcine nucleus pulposus	Adipose-derived MSC exosomes	Intervertebral disc degeneration treatment	[215]
Pluronic F127, dECM	Human nucleus pulposus	Human MSC-derived small extracellular vesicles	Intervertebral disc regeneration treatment	[216]
GelMA, HA-DA, OHA, dECM	Porcine knee cartilage and cancellous bone	Rat BMSC exosomes	Cartilage and subchondral bone regeneration	[217]
Gelatin, quaterinized chitosan, dECM	Porcine skin	Human adipose MSC exosomes, nano-hydroxyapatite	Osteogenesis and vascularity regeneration	[218]
dECM	Small intestinal submucosa	Rat BMSC exosomes, mesoporous bioactive glass	Wound healing	[219]
HA, dECM	Rat brain tissue	TNF-α/IFN-γ- primed human umbilical cord MSC-derived extracellular vesicles, polydeoxyribonucleotide	Repair of spinal cord injury	[220]
PLGA, dECM	Porcine kidney	TNF-α/IFN-γ-primed umbilical cord MSC-derived extracellular vesicles, polydeoxyribonucleotide, magnesium hydroxide	Kidney regeneration	[221]

bFGF, basic fibroblast growth factor; BMP-2, bone morphogenetic protein-2; BMP-7, bone morphogenetic protein-7; BMSC, bone mesenchymal stem cells; GelMA, gelatin methacryloyl; HA, hyaluronic acid; HA-DA, dopamine-conjugated hyaluronic acid; HA-MA, hyaluronic acid methacryloyl; HGF, hepatocyte growth factor; IFN-γ, interferon-γ; MSC, mesenchymal stem cells; IGF-1, insulin growth factor-1; OHA, oxidative hyaluronic acid; PCL, polycaprolactone; PEGDA, polyethylene glycol diacrylate; PGCL, poly(glycolide-co-caprolactone); PLCL, poly(l-lactide-co-caprolactone); PLGA, polylactic acid-co-glycolic acid; PLLA, poly-l-lactic acid; TGF-β1, transforming growth factor-β1; TGF-β3, transforming growth factor-β3; TNF-α, tumor necrosis factor-α; VEGF, vascular endothelial growth factors.

### Growth factors

The supplementation of growth factors in the scaffold could promote tissue regeneration by promoting cell proliferation and differentiation [151]. In recent research, different kinds of growth factors, including basic fibroblast growth factor (bFGF), TGF-β superfamily, vascular endothelial growth factors (VEGF) and other types of growth factors were incorporated into dECM-based materials [152–154].

#### Basic fibroblast growth factor

As the physiological activities in organogenesis and tissue homeostasis, bFGF has been used to combine with dECM to build the composite scaffold [155]. For instance, Lin *et al*. [156] dispensed bFGF in the dECM hydrogel for central nervous system disease treatment, showing sustained bFGF release behavior in 3 days to enhance the neuroprotective effect. Liu *et al*. [157] fabricated a dECM and chitosan-based hydrogel with bFGF for supporting annulus fibrosus-derived stem cell growth. Compared with the dECM/chitosan hydrogels, after culture with the hybrid hydrogels that contained bFGF, the expressions of COL-I, COL-II and aggrecan in stem cells were significantly enhanced. In particular, Zhang *et al*. [158] designed a miniaturized porous PCL chamber by 3D printing and filled it with bFGF-loaded adipose tissue dECM hydrogel. This 3D-printed composite scaffold showed a highly cumulative bFGF release rate in 14 days, supporting the generation of new adipose tissue *in vivo*. Magnetic resonance imaging and histological results of the chamber revealed that the bFGF-loaded 3D scaffold significantly enhanced the large volume of adipose tissue regeneration and showed adipose regenerative properties for 6 mouths.

#### TGF-β superfamily

TGF-β superfamily has been widely used in tissue engineering because they could stimulate multiple cell functions, including TGF-β1/2/3 and bone morphogenetic proteins (BMPs) [159]. To this end, several studies applied the TGF-β superfamily to design multifunctional systems combined with dECM [160]. For example, Larochette *et al*. [161] developed bone morphogenetic protein-2 (BMP-2)-incorporated osteogenic-differentiated MSC-derived dECM scaffolds for ectopic bone formation. Tan *et al*. [162] impregnated BMP-2 into dECM hydrogel, aiming to achieve bone defects repair in rat model. *In vivo* results demonstrated that the BMP-2-loaded dECM hydrogel could modulate macrophage polarization and accelerate bone regeneration of promoting angiogenesis and osteogenesis.

**Figure 8.**
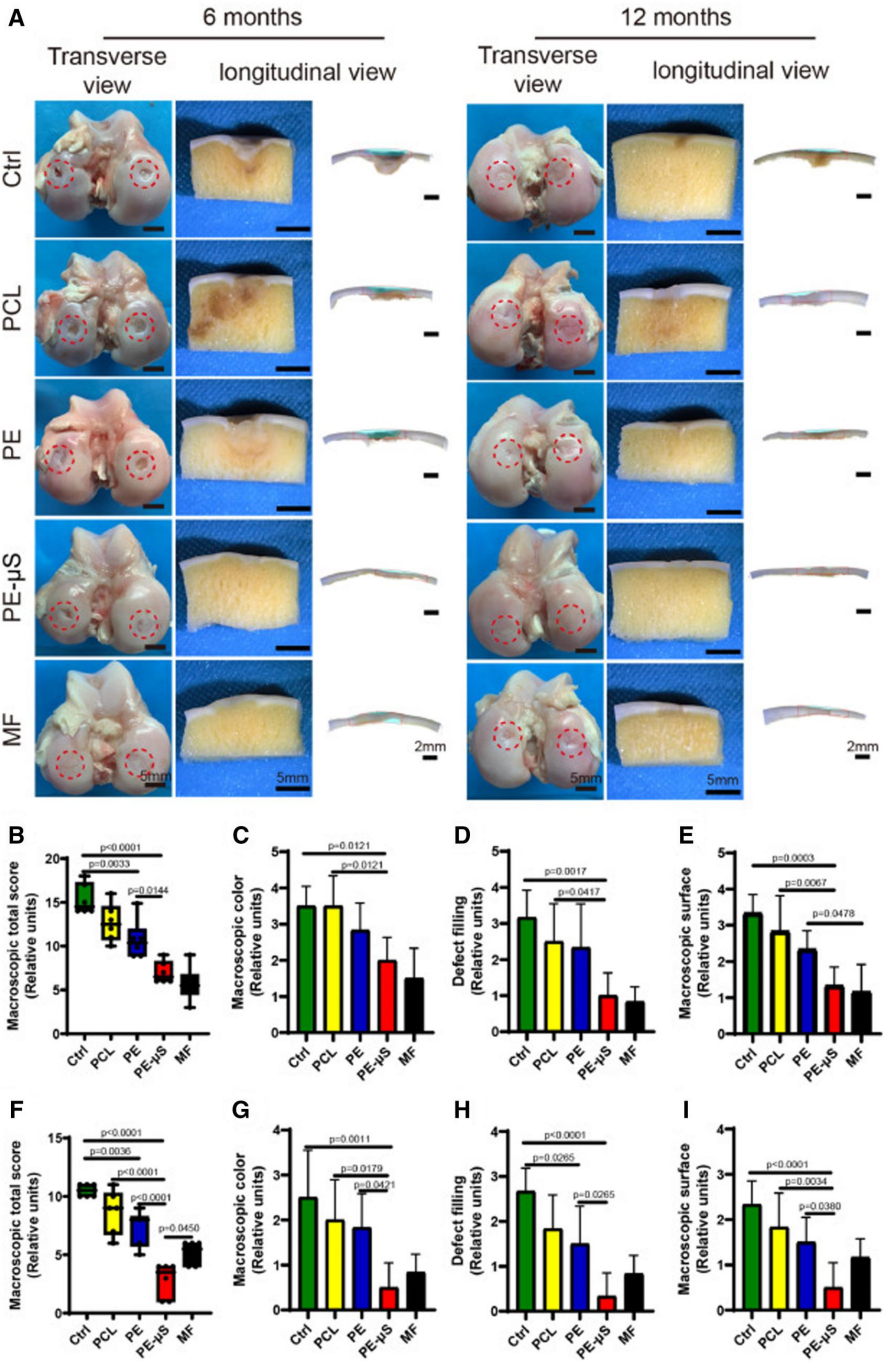
(**A**) Representative macroscopic images of the repaired tissues. (**B**) Macroscopic total scores, (**C**) macroscopic color, (**D**) defect filling (**E**) and macroscopic surface at 6 months. (**F**) Macroscopic total scores, (**G**) macroscopic color, (**H**) defect filling and (**I**) macroscopic surface at 12 months. Adapted with permission from Ref. [165].

In another study, Wei *et al*. [163] fabricated a multifunctional system made by polyethylene glycol diacrylate (PEGDA) and annulus fibrosus dECM capable of continuously releasing TGF-β1 in 7 days. Compared with other groups, the TGF-β1-loaded injectable hydrogel promoted the migration of annulus fibrosus cells, enhancing ECM deposition to alleviate disc degeneration. Zhang *et al*. [164] prepared silk fibroin–dECM bioink encapsulated with TGF-β3 by 3D printing for cartilage tissue engineering. The hybrid bioink with suitable mechanical strength for cell growth could control the release of TGF-β3 for chondrogenic differentiation promotion. Similarly, Yang *et al*. [165] developed PLGA microspheres to control the TGF-β3 releasement and then loaded them with a functional cartilage scaffold based on PCL, dECM and GelMA. *In vitro* analysis indicated that TGF-β3-loaded microspheres containing bioink improved the MSC migration and chondrogenic differentiation. Furthermore, in a full-thickness articular cartilage defects sheep model, this composite hydrogel showed a well-organized collagen orientation, indicating the cartilage regeneration properties (Figure 8).

In addition, Zhang *et al*. [166] designed a silk fibroin and dECM composite scaffold as an integrated 3D platform to deliver TGF-β and BMP-2 to achieve osteochondral regeneration. Han *et al*. [167] incorporated TGF-β1, BMP-2 and insulin growth factor-1 (IGF-1) into PEGDA–dECM composite scaffold using high-precision 3D printing technology. In this platform, the TGF-β1 and BMP-7 loaded hydrogel was acted as the upper layer to reduce cartilage surface friction. In contrast, the downer layer contained TGF-β1 and IGF-1, promoting the production of collagen and proteoglycans. The multiple growth factors loaded hybrid scaffold could successfully guide cartilage regeneration by promoting chondrogenic differentiation and alleviating the inflammatory response.

#### Vascular endothelial growth factor

VEGF, an essential growth factor regulating neovascularization and angiogenesis, has also been processed into a dECM-based scaffold for tissue regeneration [168]. Liu *et al*. [169] found that the combination of dECM and VEGF hydrogel could enhance tube formation and accelerate wound healing process. In another case, Liu *et al*. [170] combined SF membrane encapsulating different growth factors (TGF-β1 and VEGF) with dECM membrane as a composite vascular graft by coaxial aqueous electrospinning. The combination of dual growth factors in one membrane was used for promoting endothelialization and preventing collagen over-deposition, indicating the potential for vascular tissue engineering applications. Hwang *et al*. [171] designed a new generation of 3D dECM-based scaffolds to support tissue regeneration that explores the incorporation of VEGF and FGF. In detail, the outer layer of the 3D patch was made by VEGF-laden HA-MA and dECM ink, in which the layer contained hepatocyte growth factor (HGF)-laden HA-MA and dECM inks. Due to the sequential and sustained release of VEGF and HGF, this patch significantly promoted angiogenesis and induced neovascularization in the brain after implantation.

### Small molecular drugs

By adding small molecular drugs with special functions into scaffolds, the hybrid scaffold can be used to achieve drug release in demand to improve the activities of drugs [172]. Several types of scaffolds composed of dECM and small molecular drugs were recently designed to accelerate tissue repair and regeneration.

#### Antibacterial drugs

**Figure 9.**
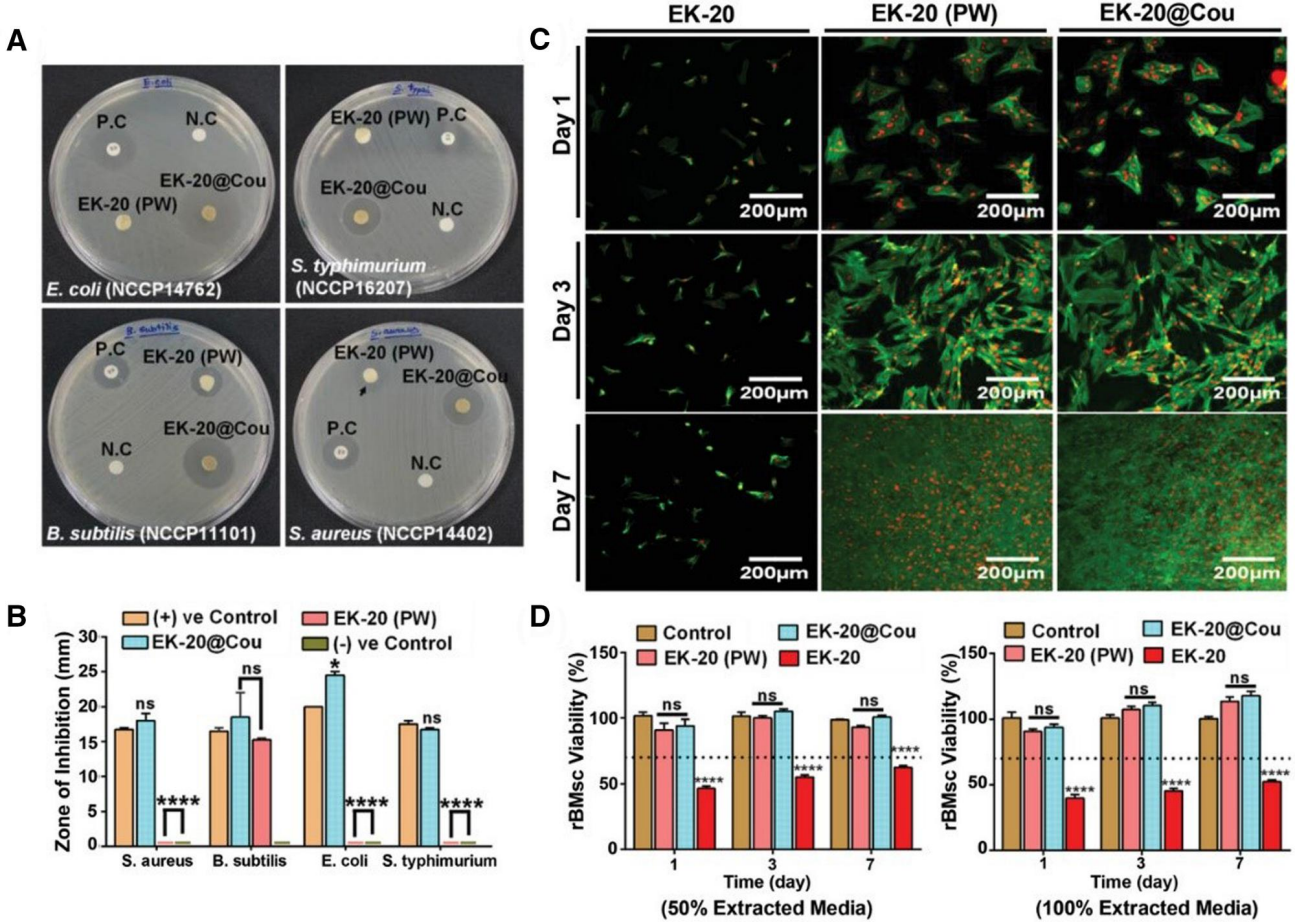
(**A**) Representative optical images of the zone of inhibition against bacteria. (**B**) Quantitative analysis of zone of inhibition. (**C**) Confocal images of the rBMSC cells. (**D**) The rBMSCs viability in different scaffolds. Adapted with permission from Ref. [175].

Although dECM-based materials are widely studied as wound dressings, eradicating bacterial infections is crucial in wound healing. Following the previous research, antibacterial agents were loaded onto dECM to fabricate new-generation dressings [173]. For instance, Chandika *et al*. [174] mixed usnic acid with PCL and dECM to develop novel composite nanofibrous using electrospinning technology. Arin *et al*. [175] used decellularized kelp as crosslinking material to strengthen the physical structure of liver dECM and then loaded it with p-coumaric acid (EK-20). Compared with the control group, the composite wound dressing achieved superior wound healing *in vivo*. On the 14th day, the wounds of the EK-20 group with 98.75% of wound closure percentage, while the wounds of the control group showed 26.82% wound closure rate (Figure 9). Additionally, the immuno-histological and biochemical phenotyping of macrophages results concluded that the released p-coumaric acid could regulate the immune response and switch the macrophages from M1 to M2 phenotype.

To achieve sustained drug release, voriconazole-loaded PLGA microspheres were produced by using emulsion evaporation technique before being mixed with dECM and gelatin composite hydrogel [176]. The drug-loaded hybrid hydrogel showed excellent antifungal properties in a rabbit corneal defect model, which could prevent fungal infection and promote corneal regeneration. Similarly, Liu *et al*. [177] fabricated polydopamine nanoparticles loaded with asiaticoside and mixed with dECM and GelMA composite hydrogel, which has the potential for scarless wound healing application. Li *et al*. [178] functionalized bovine pericardia dECM scaffold with copper@tea polyphenol self-assembly nanoparticles based on the coordination of tea polyphenol with copper ions. As expected, the scaffold maintained the antimicrobial activity of nanoparticles, which also exhibited anti-calcification, remodeling and integration properties for cardiovascular applications.

#### Other drugs

Apart from the antibacterial drugs, other drugs, such as anti-resorptive agents and antioxidants, among others, were also combinated with dECM-based scaffolds in biomedical applications, including bone and vascular tissue engineering, among others [179–181]. For example, Xie *et al*. [182] fabricated the nanofibers composed of dECM and PLGA-loaded pifithrin-α acting as bilayer bone scaffold for enhancing osteoinductivity. Zhou *et al*. [183] fabricated icariin-loaded poly(glycolide-co-caprolactone) microparticles with suitable porous structures and coated with BMSC-derived dECM. Subsequently, the sustained release of the icariin from the microcarriers showed excellent synergistic effects with dECM in the rat models. In another study, Datta *et al*. [184] designed alendronate-loaded gelatin microspheres embedded in bone dECM/oleoyl chitosan hydrogel for loading BMP-2. In the rabbit tibial bone defects model, the dual drug-loaded composite hydrogel showed 76% defect closure, whereas the bone dECM/oleoyl chitosan hydrogel had a 56% defect closure rate after 4 weeks of implantation.

Inhibition of thrombus formation and intimal hyperplasia, proangiogenic, have positive impacts on enhancing the performance of vascular grafts *in vivo*. Thus, different drug agent was incorporated in dECM-based vascular grafts to achieve better performance in tissue regeneration [185, 186]. Gao *et al*. [187] produced atorvastatin-loaded PLGA microspheres for delivering endothelial progenitor cells and mixed with dECM and alginate-based bioink to construct bio-blood-vessel. The cumulative release of atorvastatin constantly improved the neovascularization of endothelial progenitor cells and avoided the risk of overdose, exhibiting synergistic therapeutic effects on mice’s hind limb ischemia model. Yang *et al*. [188] fabricated vascular grafts with small diameter that consisted of rapamycin-loaded PCL nanofibrous as an outer layer to coat on the rat aorta dECM layer. The novel hybrid vascular grafts significantly reduced neo-intimal hyperplasia after abdominal aorta transplantation at 12 weeks. Shi *et al*. [189] developed a tri-layered vascular graft *via* electrospun by sandwiching media dECM powders loaded in PLCL between outer layers that loaded intima powders and inner layers that salidroside. In this platform, the salidroside of the inner layer inhibited thrombus formation and reduced the adhesion effect of platelets, whereas the dECM powder significantly promoted smooth muscle regeneration and endothelialization.

### Inorganic particles

Inorganic particles have shown unique properties in medical applications, which have emerged as one of the probable agents in tissue regeneration [190]. Adding the inorganic particles into biomaterials could remedy the mechanical shortcomings and increase their bioactivity for tissue regeneration [191]. Thus, to integrate both advantages of inorganic particles and dECM, the dECM–inorganic particles composite scaffold with outstanding mechanical properties and multi-functionalities have been developed.

#### Hydroxyapatite

Hydroxyapatite is widely used as an inorganic material for bone regeneration due to its similar mineral composition to bone. Researchers developed lots of dECM/hydroxyapatite composite scaffolds by different manufacturing processes, achieving a better bone repair effect than using those materials alone [192, 193]. Parmaksiz *et al*. [194] prepared a multilayer composite scaffold using small intestinal submucosa dECM as layer and PCL-hydroxyapatite microparticles as a binder. By co-culturing with rat bone marrow MSCs, this composite scaffold showed higher osteocalcin and alkaline phosphatase expression, proving the potential for new bone formation. Hwangbo *et al*. [195] reported a hydroxyapatite-loaded PLLA–dECM bone tissue scaffold by adopting 3D printing technology. By adjusting the weight fraction of hydroxyapatite in scaffold and the particle size of hydroxyapatite, the composite scaffold obtained 96.3–136.8 MPa flexural strengths and flexural moduli around 1.4 GPa. After culturing with human adipose stem cells, Runt-related transcription factor 2 (Runx2) and alkaline phosphatase expressions in the hydroxyapatite loaded PLLA–dECM group were significantly improved than the hydroxyapatite/PLLA scaffold. In addition, Yang *et al*. [196] fabricated Sr^2+^/Fe^3+^ doped hydroxyapatite modified dECM scaffold with a rough surface and suitable mechanical strength using extrusion cryogenic 3D printing technology. The hydroxyapatite modified dECM scaffold provided a desirable microenvironment for bone regeneration, promoting the angiogenesis and osteogenesis properties of cells, enhancing biomineralization ability and manipulating the immunoregulation process.

#### Graphene-based particles

Based on the unique mechanical and electronic properties, graphene oxide functioned scaffold has attracted increased attention [197, 198]. For example, Pereira *et al*. [199] coated the graphene oxide on decellularized umbilical cord arteries to enhance mechanical performance. Compared to the dECM scaffold, the maximum force, burst pressure, strain and compliance of graphene oxide modified dECM scaffold significantly enhanced. Likely, Kim *et al*. [200] crosslinked the dECM scaffold with nano-graphene oxide to alleviate the enzymatic degradation activity and enhance the mechanical rigidity. Integrating nano-graphene oxide within dECM scaffolds has shown high stability after implantation for 60 days, alleviating the graft-elicited inflammation responses by regulating macrophage polarity. More importantly, the bioengineered scaffold showed superior hepatic regeneration activity after implantation in rat acute or chronic liver failure models.

Barroca *et al*. [201] found that incorporating reduced graphene oxide into adipose dECM scaffolds could induce neuron differentiation. In another work, Tsui *et al*. [202] developed a reduced graphene oxide-contained dECM scaffold to mimic the cardiac microenvironment. By regulating the reduction degree of reduced graphene oxide and the concentration in the hydrogel, the mechanical properties and conductivity of scaffolds could be tuned. After cultured the cardiomyocytes in this composite hydrogel, the genes associated with regulating contractile function were significantly enhanced, indicating the synergistic effect of dECM and graphene oxide reduced in tissue engineering. Zheng *et al*. [203] synthesized magnesium-enriched graphene oxide nanoparticles for modulating the inflammatory responses and then combined them with bone dECM scaffold for bone regeneration. Compared to the bone dECM scaffold, the 3 months post-implantation results showed that the magnesium-enriched graphene oxide nanoparticles-based dECM hydrogel showed significantly higher neovascularization lever.

#### Other inorganic nanoparticles

Combining the outstanding merits of other inorganic nanoparticles (nanoceria, laponite, metal or non-metal particles) with dECM, the hybrid scaffold exhibited excellent performance in tissue regeneration [204]. For example, nanoceria can be used as an antibacterial/antioxidant agent in hydrogel to offer multifunctional properties. Singh *et al*. [205] found that the activity accelerates tissue regeneration by combining nanoceria and dECM. Furthermore, they functionalized goat small intestine dECM composite scaffolds with nanoceria and nanoemulsion-loaded curcumin [206]. Additionally, the composite scaffold prolonged curcumin release with antibacterial properties and showed excellent free radicals scavenging activity, which significantly enhanced the wound healing rate.

Based on the 2D structure and special composition, Laponite has been used to enhance the mechanical properties and degradation time of dECM in tissue engineering [207]. Shin *et al*. [208] bioprinted a PEGDA and cardiac dECM hydrogel reinforced with Laponite to model cardiac tissue. In addition, some novel inorganic nanoparticles with extraordinary characteristics were used to enhance the bioactivity of dECM. In recent, Shen *et al*. [209] designed a black phosphorus nanosheets degradation loaded photothermal scaffold based on fish scale dECM and GelMA to accelerate bone regeneration. Combined with the photothermal therapy, the photothermal scaffold promoted osteocalcin accumulation and facilitated angiogenesis. Jin *et al*. [210] developed a synergistic platform based on copper oxide nanoparticles-loaded PLGA nanofibers and dECM hydrogel with human adipose-derived MSC. In this system, copper oxide nanoparticles with reactive oxygen species elimination ability significantly promoted cell migration and proliferation. Moreover, the copper oxide nanoparticles loaded in nanofiber-reinforced hydrogel could promote liver regeneration and benefit liver function recovery, which showed outstanding synergistic effects in acute liver failure treatment.

### Extracellular vesicles

Extracellular vesicles, a kind of paracrine factor obtained for cells, contain proteins, nucleic acids and growth factors. Due to the biological activities, the bioactive properties of extracellular vesicles have been used to cooperate with scaffolds in tissue regeneration [211–213]. To combine the exosomes and dECM for achieving tissue repair and regeneration, Wang *et al*. [214] designed fish skin dECM hydrogel to bind to BMSCs, leading to a stronger synergistic effect on promoting bone regeneration. Xing *et al*. [215] innovatively incorporated adipose-derived MSC-exosomes into dECM hydrogels to modulate the microenvironment of intervertebral disc degeneration. In this system, exosomes could accumulate the ECM in the microenvironment by regulating matrix metalloproteinases and inhibit pyroptosis by decreasing the expression of inflammatory factor. Meanwhile, the dECM-based injectable hydrogel provided a tissue-specific environment for nucleus pulposus cell growth. According to the result of the tail vertebral disc degeneration model, this composite hydrogel exhibited higher levers of aggrecan and collagen than other groups, which provided a promising strategy for intervertebral disc degeneration treatment.

**Figure 10.**
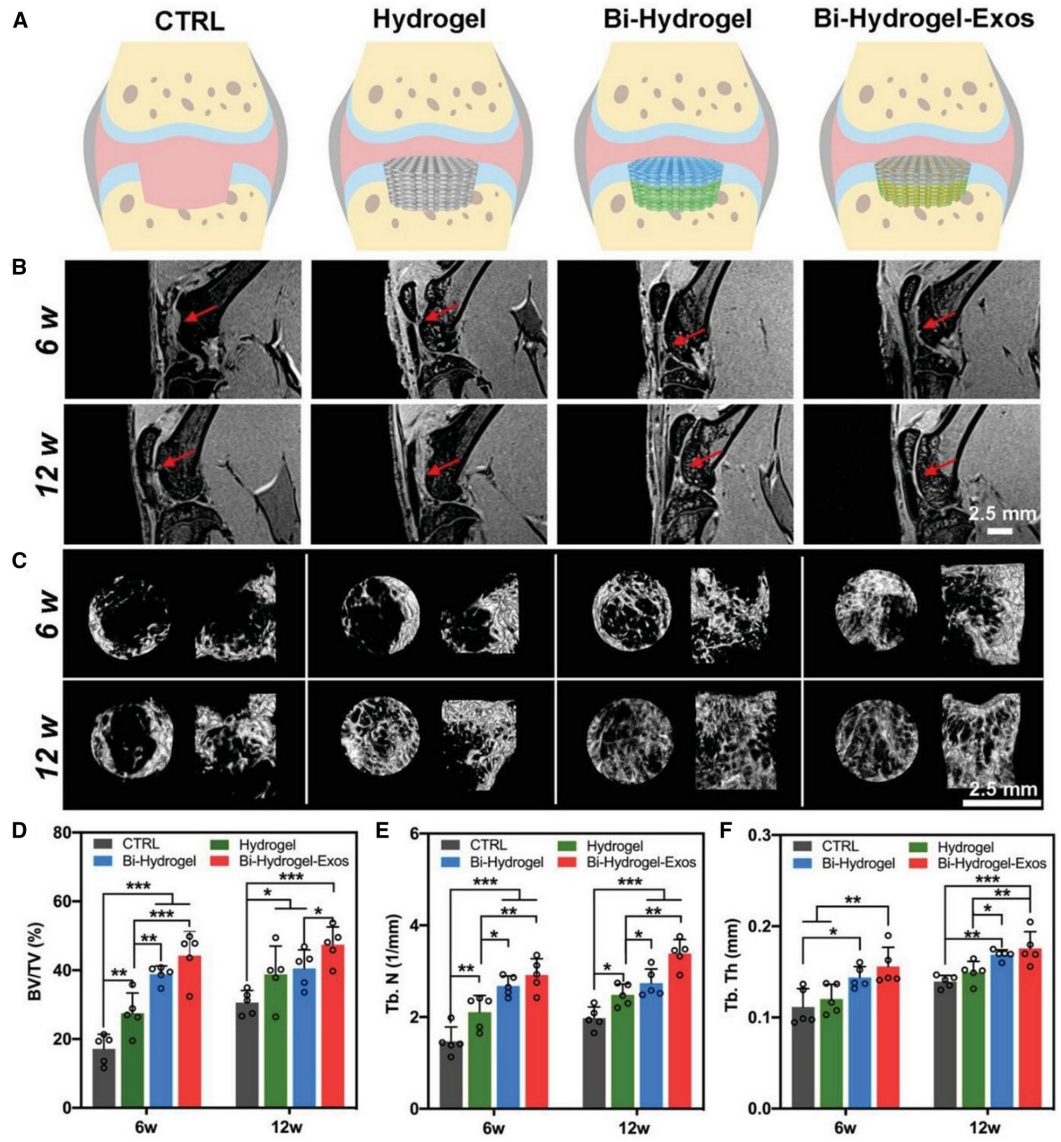
(**A**) An overview illustrating 3D-printed different scaffolds implanted in rat knee osteochondral defects. (**B**) Representative MRI images. (**C**) Representative 3D reconstructed micro-CT images at defect sites. Quantitative analysis of (**D**) bone volume/total volume, (**E**) trabecular number and (**F**) trabecular thickness for regenerated bone tissues in the defects. Adapted with permission from Ref. [217].

For achieving sustained release of extracellular vesicles, Liao *et al*. [216] fabricated a thermo-responsive hydrogel that composed of Pluronic F127 and nucleus pulposus dECM for human MSC-derived extracellular vesicle delivery. Additionally, Li *et al*. [217] designed a 3D double-network hydrogel by using GelMA, oxidative HA, dopamine-conjugated HA, cartilage dECM and bone dECM as raw materials, encapsulating with human adipose MSC exosomes. This composite system showed 80% exosome release efficiency in 24 days. After being implanted in a rat model for 12 weeks, the spatial microenvironment-biomimetic 3D printed scaffolds filled with cartilage-defected tissue and displayed higher trabecular thickness (Figure 10). In addition, *in vivo* results have shown that his combined 3D hydrogel-exosomes significantly reduced inflammatory reaction by inflammatory factor interleukin-1β expression.

In addition, extracellular vesicles were combined with other bioactive factors in dECM-based scaffold forms, which exerted synergistic effects in tissue engineering [218]. Hu *et al*. [219] designed a 3D scaffold based on dECM, mesoporous bioactive glass and exosomes by cryogenic printing technology. The exosomes-loaded composite scaffold exhibited hemostasis ability in the liver hemorrhaging model and enabled skin regeneration in 14 days in the diabetic wound healing model. Roh *et al*. [220] incorporated MSC-derived extracellular vesicles and polydeoxyribonucleotide into HA and dECM-based scaffold, studying its spinal cord regeneration activity. In this system, combining extracellular vesicles and polydeoxyribonucleotide reduced inflammation in the tissue microenvironment during repair and promoted angiogenesis, axonal regrowth and anti-apoptosis of neurons. *In vivo* study demonstrated that integrating exosomes and polydeoxyribonucleotide conduits was a useful therapeutic intervention in treating acute or chronic spinal cord injury. Likely, Ko *et al*. [221] integrated the biochemical cues of exosomes and polydeoxyribonucleotide into PLGA-based scaffolds containing magnesium hydroxide and kidney dECM. Moreover, the multifunctional scaffold system provided an optimal microenvironment for kidney regeneration promotion.

## Conclusion and outlook

dECM has gained popularity in tissue regeneration applications due to its biochemical and biophysical components from native tissues, which can mimic the complexity of organs and excellent biocompatibility. Moreover, the dECM solution tended to form 3D hydrogel at 37°C based on the thermal crosslinking mechanism, which provided a tissue-specific structure to support cell growth and remodel the tissue microenvironment. Nonetheless, the poor mechanical properties and rapid degradation of pure dECM hydrogel remain an inherent challenge for further application. Fortunately, the multiphasic designs of dECM-based biomaterials, including additive bioactive factors and integration of other biomaterials in a multifunctional system, have enhanced the preclinical tissue regeneration outcomes of dECM. In this article, we summarized the recent researches in designing and fabricating dECM-based composite scaffolds (nature polymers, synthetic polymers, bioactive factors) and their applications in regenerative medicines. Although designing multifunctional dECM-based composite materials is an essential research area in tissue engineering, several challenges remain in building integrated dECM-based composite scaffolds for successful clinical translation.

Along with the charming development of decellularized methods recently, dECM from different organs was obtained by physical, chemical, biological and a combination of these techniques. The agents used in traditional decellularized processes would induce potential toxicity, which may be an essential challenge in the future. Moreover, the unstable dECM yield, unavoidable bioactive factor loss and heterogeneity between different batches still need to be addressed. Due to the lack of standard protocol and infrastructure, the high production cost and the time-consuming dECM production are unavoidable problems faced in the industrial scale-up. Therefore, the researchers should focus on developing novel decellularized methods to rapidly obtain dECM that maintain a high degree of bioactive factors in different tissue/organs.

Despite the outstanding functional reconstruction potential of dECM-based scaffolds, choosing suitable sterilization methods after decellularization is still an existent challenge that hindered the clinical applications of dECM. Recently, researchers demonstrated several sterilization and disinfection methods, such as ultraviolet irradiation, ethylene oxide and supercritical carbon dioxide. However, due to the different compositions and 3D structures of native tissue or organs, those sterilization methods may not be suitable for obtaining different kinds of dECM. Therefore, it is undoubtedly a great research future direction to develop appropriate sterilization and disinfection methods thought over synthetically the properties and structure of various dECMs.

Clinical results on dECM-based commercial products suggested its great potential in tissue regeneration. Thus, the clinic application of dECM-based composite scaffolds is meaningful but challenging. Although emerging research focused on the tissue generation activity of dECM-based composite scaffolds, the long-time biosafety and potential toxicity of these composite scaffolds rarely receive attention. As the degradation products of adding polymers or bioactive factors might influence the tissue regeneration microenvironment, the current preclinical studies are insufficient for supporting the successful clinical application of dECM-based composite scaffolds. As a result, researchers should consider the clinical use and potential toxicity to humans while designing dECM-based composite systems for tissue regeneration.

Despite the significant development, there exist several scientific challenges hindering the clinical application of dECM-based composite scaffolds. The animal trials of dECM-based composite scaffolds have been widely investigated on small animal models, such as mouse or rat models, lacking comprehensive investigations of their therapeutic effects on large animal models. More importantly, even beagle models and goat models showed similar implant sizes to humans, and those models were not suitable for replicating the physiological environment and tissue forces of humans. In addition, the scaling up production of scaffolds remains a challenge for the clinical application of dECM-based composite scaffolds.

An engineered composite scaffold made of dECM or combined with other polymers can be useful for delivering various therapeutic agents to achieve tissue regeneration. However, tissue regeneration is a dynamic and complex process that involves cell recruitment, adhesion, proliferation, differentiation, ECM formation and remodeling. Although single growth factors and multiple growth factors enhanced the biological functions of dECM, the unstable bioactivity and burst release of growth factors still hinder the medicine application. Therefore, the addition of exosome and platelet-rich plasma obtained from the body in a dECM-based scaffold should be considered. Moreover, combining different agents has shown synergistic effects in tissue regeneration promotion. Thus, the fabrication of dECM composite scaffolds with on-demand drug delivery and multifunctional and controllable shapes will become a research hot point in the future.

In conclusion, despite the remaining problems, tissue engineering using dECM-based multiphasic scaffolds is an ideal and effective strategy for tissue defect repair in clinical applications. With the cross-disciplinary development in innovative biofabrication technology, novel materials designing and cell engineering, we believe that dECM-based composite materials would be promising alternatives for achieving defective tissue reconstruction and regeneration.
